# Gradient multilayered RTV silicone rubber composites with carbonyl iron, conductive carbon, and ZnS for broadband microwave absorption and infrared stealth

**DOI:** 10.1039/d6ra07751j

**Published:** 2026-10-08

**Authors:** Li Hao, Zhao Shuwei, Tu Qunzhang, Shen Xinmin, Peng Wenqiang, Liu Qing, Yin Qin, Ma Yao

**Affiliations:** a Army Engineering University Nanjing Jiangsu 210007 China xmshen2014@aeu.edu.cn; b National University of Defense Technology Changsha Hunan 410073 China pengwenqiang08@nudt.edu.cn

## Abstract

The integration of broadband microwave absorption, infrared modulation, and mechanical flexibility within a single material remains a formidable challenge. Herein, a gradient multilayered composite is constructed by sequentially casting condensation-type room-temperature-vulcanized silicone rubber (RTV) layers containing carbonyl iron powder (CIP), conductive carbon black (CB), and zinc sulfide (ZnS) at progressively increasing filler loadings. This architecture spatially decouples impedance matching, electromagnetic attenuation, conductive reflection, and infrared modulation. The gradient composite achieves an absorption coefficient exceeding 0.9 across 8–18 GHz, corresponding to a 10 GHz effective absorption bandwidth. This broadband performance originates from a gradient impedance matching mechanism, whereby the top low-permittivity layer admits microwaves with minimal reflection and the underlying CIP layers progressively attenuate the transmitted energy through magnetic and dielectric losses, while the conductive CB bottom layer redirects residual waves back into the lossy region for a second attenuation pass. The mechanism is verified by transmission matrix method calculations and full-wave electromagnetic simulations. The ZnS-containing layer imparts tunable infrared emissivity, decreasing from 0.781 to 0.695 (3–5 µm) as the ZnS content increases from 10 to 30 wt%, with a steady-state radiation temperature reduction of 22.6 °C relative to Neat RTV. Mechanically, the gradient composite retains an elongation at break of 73.8%, approximately 2.6 times that of the individual brittle functional layers, owing to the protective effect of the ductile outer layers. This gradient multilayered design overcomes the inherent trade-offs among microwave absorption, infrared modulation, and mechanical flexibility, offering a versatile platform for flexible multifunctional composites.

## Abstract 2

The integration of broadband microwave absorption, infrared modulation, and mechanical flexibility within a single material remains a formidable challenge.

## Graphical abstract

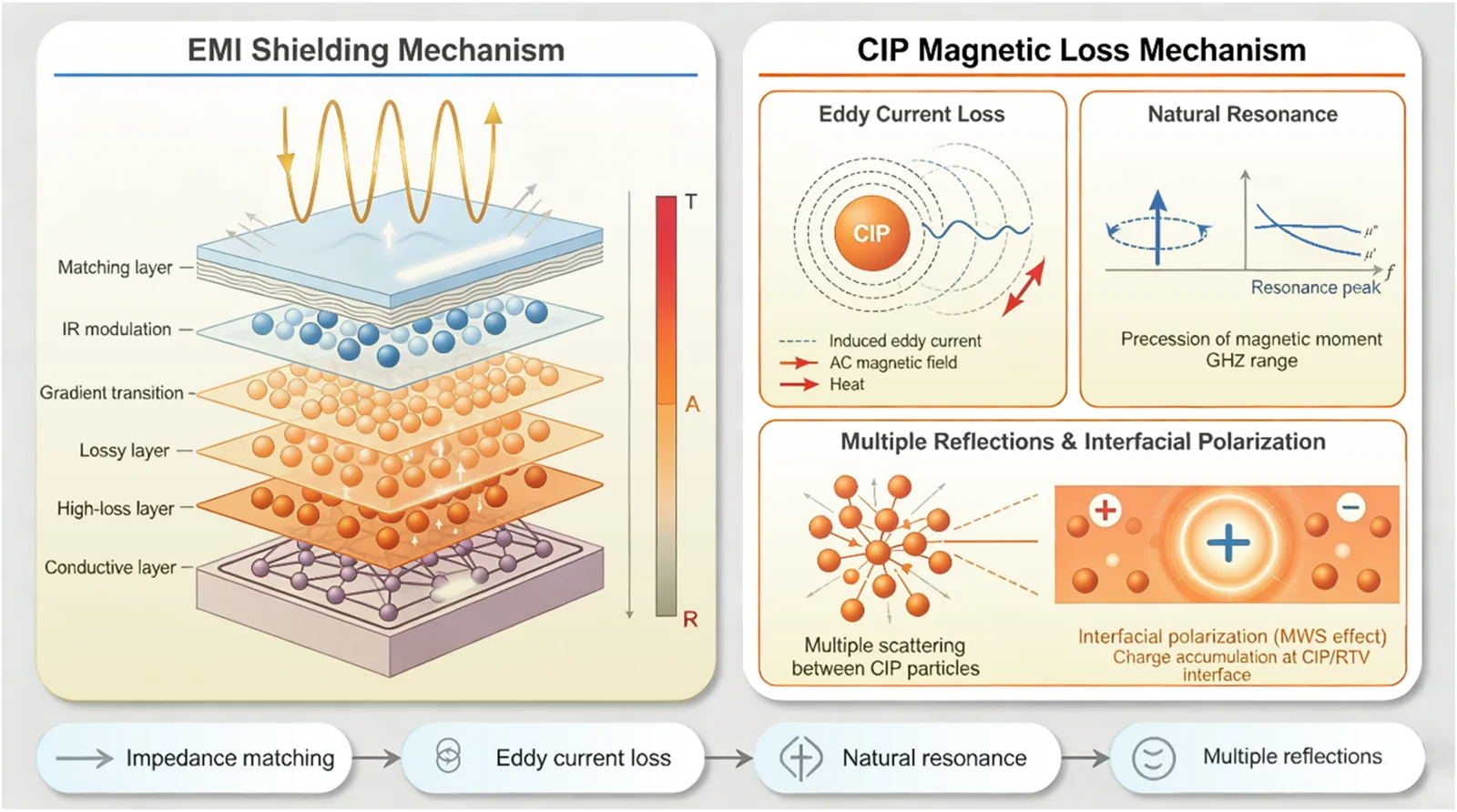

## 1. Introduction

The rapid proliferation of 5G wireless communication, wearable electronics, and advanced radar systems has led to increasingly complex electromagnetic environments, driving an urgent demand for high-performance microwave absorbing materials (MAMs) that can mitigate electromagnetic pollution and reduce radar cross-sections.^1–3^ Concurrently, the expanding deployment of infrared (IR) sensors across diverse settings, spanning from non-contact body temperature monitoring to thermal management in intelligent buildings, has intensified the need for materials that can precisely tune thermal radiation.^4,5^ In wearable health-monitoring devices, for instance, the IR signature of electronic components must be carefully managed to prevent thermal crosstalk that would otherwise distort sensor readings.^6^ These converging demands call for a flexible material platform that simultaneously delivers broadband microwave absorption, IR modulation, and adequate mechanical robustness. Integrating these three functionalities into a single material system remains challenging, because conventional metallic absorbers, despite their high attenuation effectiveness, are intrinsically dense, rigid, and prone to oxidation — properties that severely limit their use in scenarios demanding lightweight construction and multispectral compatibility.^7,8^ This limitation has consequently spurred growing interest in flexible polymer-matrix composites that synergistically combine broadband microwave absorption and IR modulation functions.^9,10^

The pursuit of high-performance microwave absorption and IR modulation has stimulated extensive exploration of functional fillers and structural designs. On the microwave absorption front, MXenes have attracted considerable attention owing to their exceptional conductivity and multi-layered architectures that facilitate interfacial polarization and multiple internal reflections.^11–13^ Their scalable application, however, is constrained by complex synthesis protocols and oxidative instability under ambient conditions.^14^ Carbon-based nanofillers, including reduced graphene oxide and carbon nanotubes, enable efficient conductive network formation at low percolation thresholds, yet their intrinsically high permittivity frequently results in severe impedance mismatch and narrow effective absorption bandwidth.^15,16^ Magnetic ferrites such as Fe_3_O_4_ and CoFe_2_O_4_ provide appreciable magnetic loss and have been widely investigated as microwave absorbers,^17^ while their high intrinsic density, limited dielectric loss, and insufficient bandwidth restrict their utility in lightweight, broadband scenarios.^18^ On the IR modulation front, low-emissivity metallic coatings effectively suppress thermal radiation but are intrinsically opaque, mechanically rigid, and highly reflective across the microwave band, precluding multispectral integration.^19,20^ Beyond filler selection, structural engineering has emerged as a promising strategy to overcome the limitations of homogeneous composites. Multilayer architectures that pair an impedance-matching layer with a lossy layer have been demonstrated to mitigate surface reflection and extend the effective absorption bandwidth.^21,22^ Gradient structures with progressively increasing filler content further enhance broadband absorption through gradual impedance transition.^23,24^ Despite these encouraging advances, the simultaneous integration of broadband microwave absorption and IR modulation within a single gradient architecture has rarely been reported. The fundamental question of how electromagnetic waves are progressively attenuated across such multilayered structures remains inadequately addressed in existing studies. Moreover, the mechanical properties of these composites, which are often compromised by high filler loadings and weak interlayer adhesion, are seldom systematically evaluated. This gap between multifunctional design and structural reliability represents a critical bottleneck that must be overcome for practical applications.

Room-temperature-vulcanized silicone rubber (RTV) has been widely deployed across healthcare, electronics, and industrial sectors owing to its inherent flexibility, chemical stability, and weather resistance (Fig. 1).^25^ Beyond these established applications, the condensation-cure RTV system possesses a unique attribute that is particularly advantageous for constructing multilayered functional architectures, namely its ability to crosslink under ambient conditions without external heating.^26^ This room-temperature curing characteristic permits the sequential deposition and curing of individual layers through a straightforward casting process, enabling each functional layer to be independently formulated and stacked. To realize the desired multifunctionality, three functional fillers were strategically distributed across distinct layers. Carbonyl iron powder (CIP) served as the primary magnetic loss-type absorber, dissipating electromagnetic energy through natural resonance and eddy current loss in the GHz range.^27^ Zinc sulfide (ZnS) functioned as the IR-modulating filler, with its content systematically varied to tune the infrared emissivity of the composite.^28^ Conductive carbon black (CB) was incorporated to form a highly conductive bottom layer that functions as a flexible built-in reflective substrate, redirecting residual microwaves that survive the gradient absorption layers back into the overlying lossy regions for a second pass of attenuation. The resulting composite features a six-layer gradient architecture, in which the filler content increases progressively from the top impedance-matching layer to the bottom reflective layer. This gradient design decouples the competing requirements of wave admission and energy dissipation into spatially separated functional zones.

**Fig. 1:**
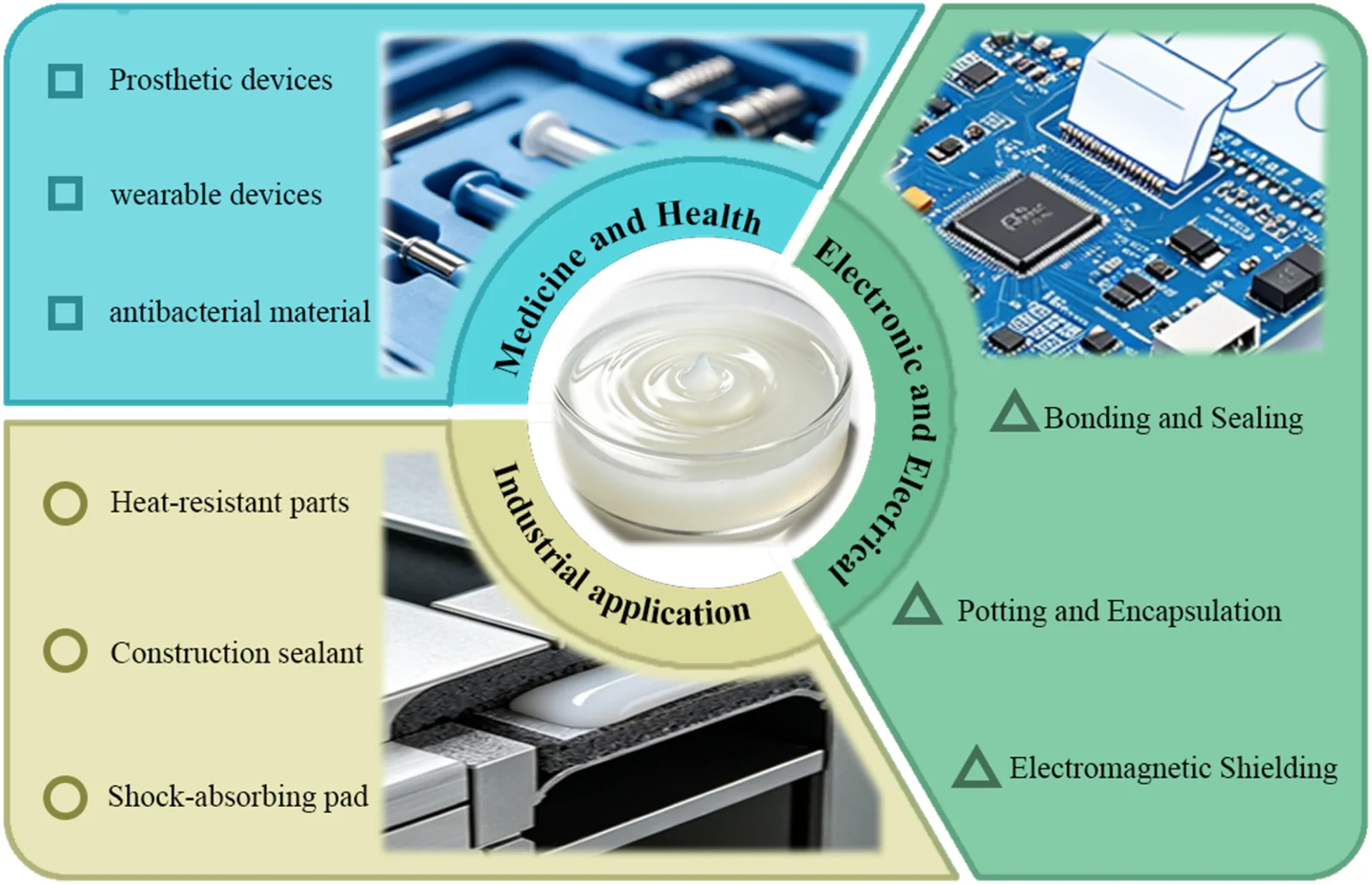
Schematic illustration of the diverse applications of RTV silicone rubber across healthcare, electronics, and industrial sectors.

In summary, this work establishes a gradient multilayered architecture within a condensation-type RTV silicone rubber system that simultaneously achieves broadband microwave absorption, tunable IR emissivity, and robust mechanical flexibility. The gradient impedance matching mechanism, elucidated through the synergistic combination of frequency-dependent electromagnetic parameter analysis, impedance matching calculations, and full-wave electromagnetic simulations, clarifies how the competing requirements of wave admission and energy dissipation can be decoupled into spatially separated functional zones. This design strategy overcomes the inherent trade-off between absorption bandwidth and mechanical integrity that has long constrained highly filled homogeneous composites. The structure–property–performance relationships established herein offer a versatile platform for the development of flexible multifunctional absorbing materials applicable to wearable electronics, thermal management, and multispectral compatibility scenarios.

## 2. Experimental

### 2.1. Materials

The condensation-type room temperature vulcanized silicone rubber was prepared from a base component (hydroxyl-terminated polysiloxane, reinforcing fillers, *etc.*) and a curing agent (polyalkyl silicate), both provided by Taiyi Advanced Materials Co., Ltd (Sichuan, China). The conductive carbon powder (TYT-900, specific surface area = 1000 m^2^ g^−1^) was purchased from Ruibo Chemical Co., Ltd (Henan, China). Carbonyl iron powder (spherical, particle size ∼3 µm) was supplied by Yibaimai Metal Materials Co., Ltd (Hebei, China). Anhydrous ethanol (C_2_H_6_O), zinc sulfide, and 3-(methacryloyloxy) propyltrimethoxysilane (KH-570) were provided by Aladdin Reagent (Shanghai, China).

### 2.2. Preparation of RTV and its composites

The RTV composite films were fabricated by a direct casting method, as schematically illustrated in Fig. 2a. For the Neat RTV film, the silicone rubber base and curing agent were mixed, mechanically stirred, degassed, poured into a mold, and cured at room temperature (25 °C). Fig. 2b depicts the underlying curing chemistry, in which hydroxyl-terminated poly(dimethylsiloxane) (PDMS-OH) undergoes a condensation reaction with tetraethyl orthosilicate (Si(OC_2_H_5_)_4_), yielding a crosslinked three-dimensional Si–O–Si network with ethanol released as a byproduct.^29^ To prepare the RTV composite films, the silicone rubber base was first mixed with CB, CIP, and ZnS at the desired mass ratios (Table 1), followed by the addition of the curing agent. All fillers were pre-treated with the silane coupling agent KH-570.

**Fig. 2:**
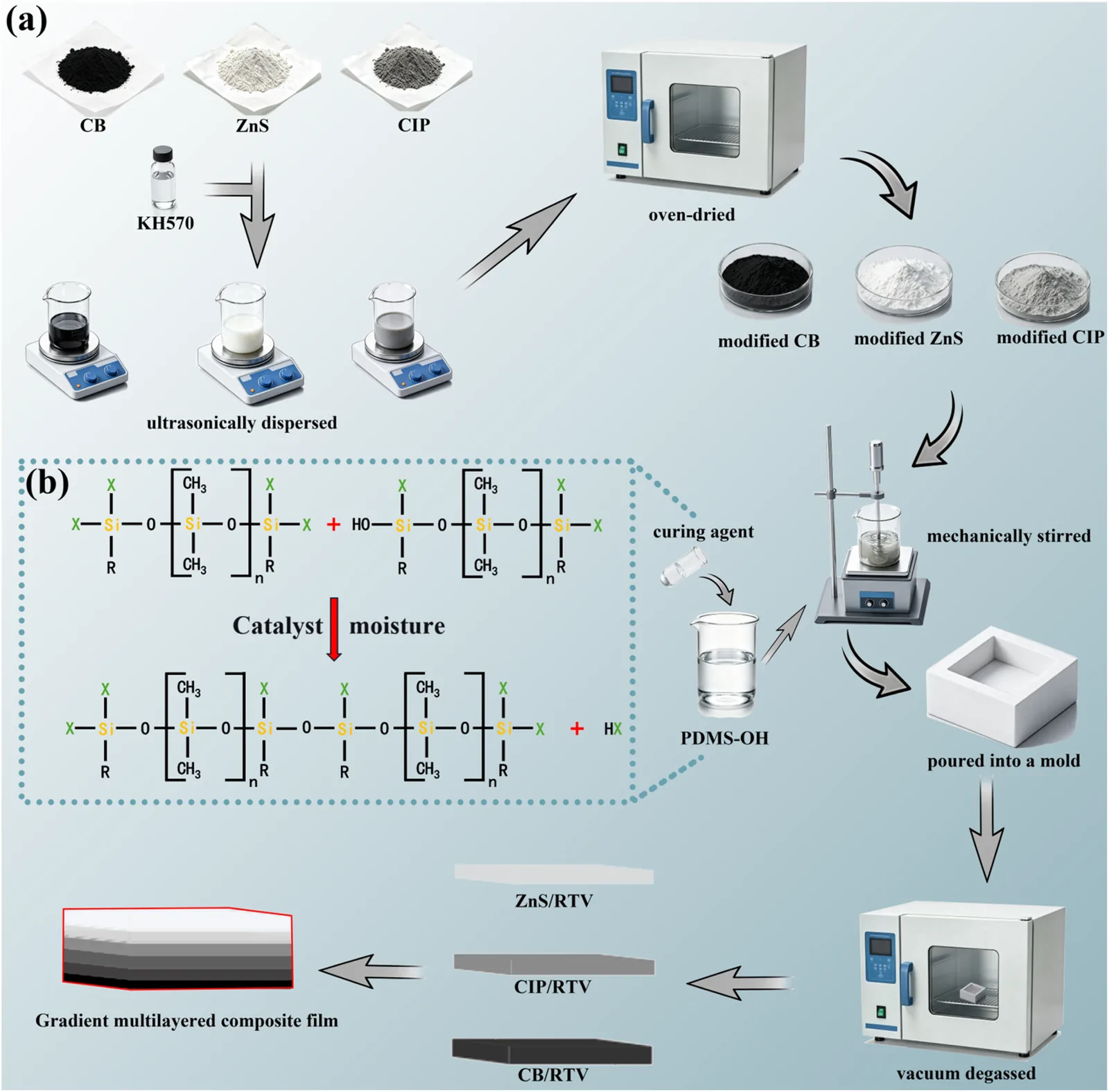
(a) Schematic illustration of the preparation of the composite films. (b) Schematic diagram of the base component (PDMS-OH) and curing agent (Si(OC_2_H_5_)_4_) during the fabricating process.

**Table 1:** Abbreviations and compositions of the prepared samples

Sample	Composition
Neat RTV	Condensation-type room temperature vulcanized silicone rubber
CIP-*x*/RTV	RTV filled with *x* wt% CIP
CB-*x*/RTV	RTV filled with *x* wt% CB
ZnS-*x*/RTV	RTV filled with *x* wt% ZnS
Neat/ZnS-*x*/CIP-*y*_1_/*y*_2_/*y*_3_/CB-*z*-RTV	Six-layer gradient composite consisting of neat RTV, ZnS-*x*/RTV, CIP-*y*/RTV, CB-*z*/RTV (from top to bottom)

Each individual layer was cast to a fixed thickness of 2 mm using a PTFE mold with a precisely machined cavity depth. For the multilayered composites, the total thickness was therefore equal to 2 mm multiplied by the number of constituent layers. For instance, the six-layer composite has a nominal total thickness of 12 mm.

### 2.3. Characterization

#### 2.3.1 FESEM

The cross-sectional morphology of the samples was observed using field-emission scanning electron microscopy (FESEM, ZEISS MERLIN Compact, Germany) at an accelerating voltage of 5.00 kV. Prior to imaging, the specimens were cryogenically fractured in liquid nitrogen and sputter-coated with a thin layer of gold to minimize charging effects. Energy-dispersive X-ray spectroscopy (EDS) coupled with FESEM.

#### 2.3.2 Reflection loss measurement

The electromagnetic parameters (complex permittivity and permeability) of the composites were measured by a vector network analyzer (VNA, Agilent E5071C, USA) using the coaxial transmission line method. Ring-shaped specimens with precise inner and outer diameters were mounted between the inner and outer conductors of the coaxial sample holder. The scattering parameters (*S*_11_, *S*_21_, *S*_12_, *S*_22_) were recorded, and the frequency-dependent complex permittivity (*ε*_r_) and complex permeability (µ_r_) were derived from the *S*-parameters using the standard Nicolson–Ross–Weir (NRW) algorithm.^30,31^ These electromagnetic parameters served as the input for the subsequent impedance matching analysis.

The reflection loss (RL) of the composites was evaluated by the arch method. For the arch method measurements, two types of specimens were prepared. Single-layer specimens with dimensions of 18 mm × 18 mm × 2 mm were used to evaluate the intrinsic absorption performance of each individual layer. The gradient multilayered composite, with dimensions of 18 mm × 18 mm × 12 mm (six layers, each 2 mm thick), was used to evaluate the absorption performance of the complete multilayer architecture. Rectangular specimens were placed on a metal backing plate, and the RL was recorded over the frequency range of 2–18 GHz using the same VNA connected to a pair of broadband horn antennas.

The RL measured in decibels (dB) by the arch method, was converted to the power reflection coefficient *R* using *R* = 10^RL/10^.^32^ Since the metal backing plate prevents transmission, the power absorption coefficient *A* is obtained as *A* = 1 − *R*. An RL value of −10 dB corresponds to *R* = 0.1, meaning that 90% of the incident microwave power is dissipated within the composite.

#### 2.3.3 Infrared measurement

The samples were placed on the temperature-controlled heating stage set at 80 °C. Their infrared thermal images were captured using an infrared thermal camera (HIKMICRO, China), and the temperature variation at the center position was recorded. The surface emissivity of the samples was measured using a handheld emissivity meter (RLK650, China) in the spectral bands of 3–5 µm and 8–12 µm. Prior to measurement, a reference standard was used for calibration to eliminate errors caused by ambient radiation. Under constant ambient temperature conditions, the lower the surface emissivity of a material, the less infrared radiation it spontaneously emits, resulting in a lower thermal contrast with the surrounding background.^33^

#### 2.3.4 Mechanical tensile testing

The mechanical properties were assessed by uniaxial tensile testing of standardized Type H2 dumbbell specimens on a universal testing machine (UTM, Instlon3365). All measurements were performed at ambient temperature with a constant crosshead displacement rate of 50 mm min^−1^, and the results were derived from linear regression fitting of at least three parallel specimens.

## 3. Results and discussion

### 3.1. Morphology and structure analysis

The microwave absorption performance and mechanical properties of the RTV-based multilayered composite films are intimately correlated with their microstructure. Fig. 3a presents the cryo-fractured cross-section of Neat RTV, which exhibits a smooth and dense surface devoid of discernible structural defects. In the CIP-filled systems, a direct comparison between Fig. 3b (modified CIP-60/RTV) and Fig. 3c (unmodified CIP-60/RTV) clearlyreveals the decisive role of surface modification in interfacial bonding. In the modified composite, CIP particles are tightly embedded within the RTV matrix, demonstrating excellent interfacial compatibility and uniform dispersion. In stark contrast, the unmodified counterpart suffers from severe particle agglomeration and interfacial debonding (microvoids), which would significantly impair the interfacial stress transfer efficiency.^34^Fig. 3d–f further illustrate the influence of filler loading. As the CIP content increases from 40 to 80 wt%, the packing density of the particles rises substantially. However, at 80 wt% loading (Fig. 3f), the excessive filler fraction induces conspicuous stress concentration sites and microstructural imperfections.

**Fig. 3:**
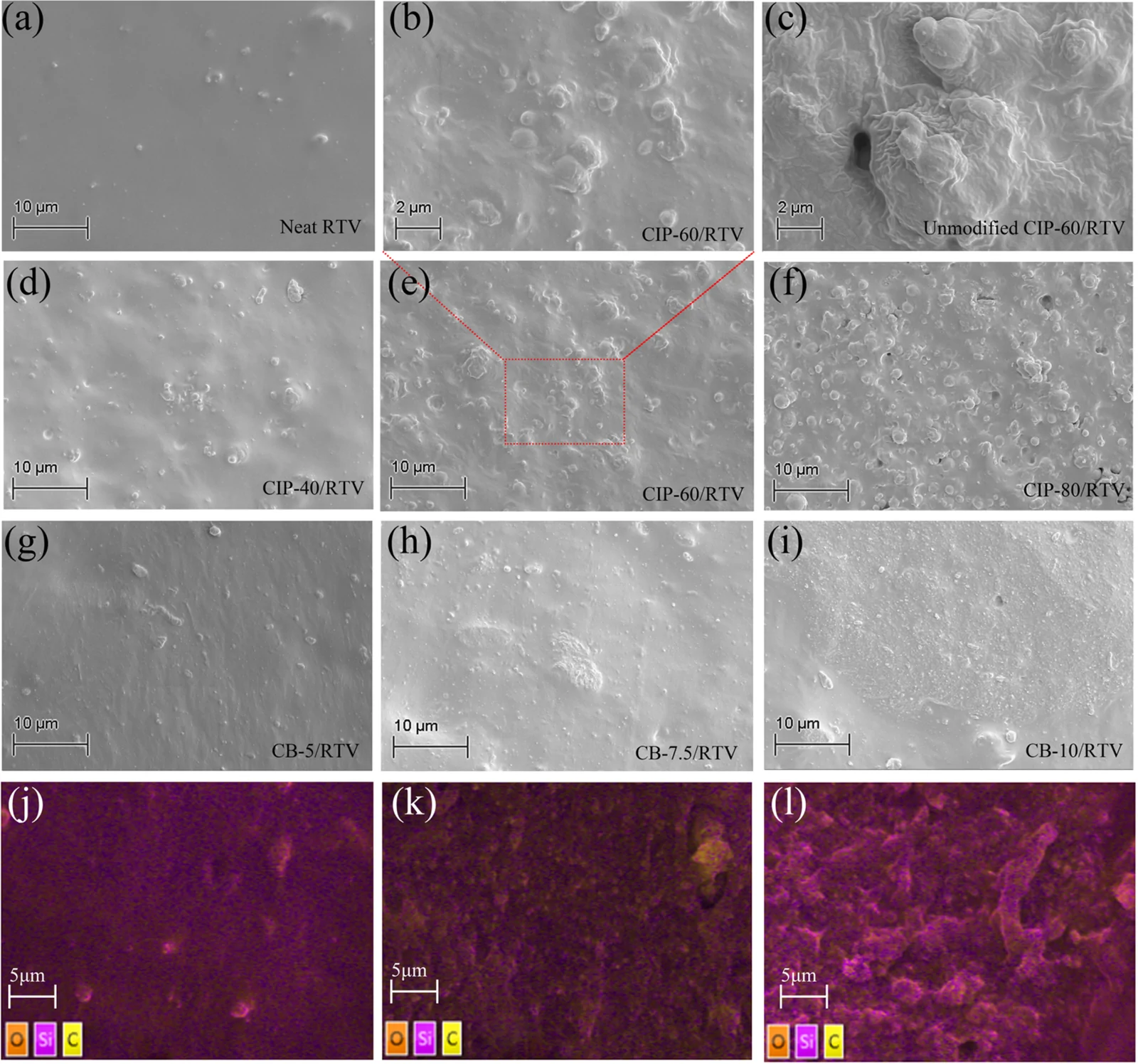
(a–i) Cross-sectional FESEM images and (j–l) EDS elemental mapping of the RTV-based composites. (a) Neat RTV at 10 µm. (b) Modified CIP-60/RTV at 2 µm. (c) Unmodified CIP-60/RTV at 2 µm. (d) CIP-40/RTV, (e) CIP-60/RTV, (f) CIP-80/RTV, (g) CB-5/RTV, (h) CB-7.5/RTV, and (i) CB-10/RTV at 10 µm. EDS layered images of (j) CB-5/RTV, (k) CB-7.5/RTV, and (l) CB-10/RTV at 5 µm.

In the CB-filled series (Fig. 3g–i), the fracture surface evolves from relatively smooth to increasingly rough as the CB content increases from 5 to 10 wt%, with the surface texture becoming progressively complex. The EDS elemental layered images (Fig. 3j–l) provide complementary evidence. The elements O (orange), Si (purple), and C (yellow) are distributed with relatively uniformity across the scanned regions. The homogeneous elemental mapping of C directly confirms that the CB fillers achieve a well-dispersed state at the macroscopic scale within the RTV matrix.

Collectively, the microscopic observations presented above establish the structural foundation for the gradient impedance matching strategy. The distinct spatial separation of the low-*ε*′ matching layers, the gradient CIP-filled attenuation layers, and the conductive CB reflective layer, as confirmed by FESEM and EDS mapping, constitutes the microstructural prerequisite for the broadband microwave absorption performance demonstrated in the following sections. These structural characteristics directly govern the filler dispersion quality and the local filler loading fraction, which in turn determine the electromagnetic parameters and impedance profile of each layer. To simultaneously achieve broadband microwave absorption and adequate mechanical resilience, it is therefore essential to optimize the filler surface modification protocol and to precisely control the filler content across the gradient architecture, thereby realizing a synergistic enhancement of the composite performance through rational microstructural design.

### 3.2. Microwave absorption mechanism

Fig. 4a presents the reflection loss of Neat RTV, CIP-40/RTV, CIP-80/RTV, CB-10/RTV (each 2 mm thick), and the gradient multilayered composite (12 mm total thickness) over 2–18 GHz. The corresponding absorption coefficients A, converted from the RL data are shown in Fig. 4b. Neat RTV exhibits a low average absorption coefficient of approximately 0.19 across the entire band, consistent with the absence of functional fillers. Single-layer CIP-40/RTV delivers an average absorption coefficient of approximately 0.27, while single-layer CIP-80/RTV reaches a higher average value of roughly 0.72. Although CIP-80/RTV possesses the strongest intrinsic loss, its effective absorption bandwidth with A exceeding 0.9 is confined to only 7.8–11.2 GHz. Single-layer CB-10/RTV exhibits a moderate absorption coefficient of approximately 0.5, reflecting partial attenuation through conductive losses. In striking contrast, the gradient multilayered composite, denoted as Neat/CIP-40/60/80/CB-10-RTV, attains an absorption coefficient exceeding 0.9 over the broad frequency span of 8–18 GHz and achieves an average absorption coefficient of approximately 0.85 over the full 2–18 GHz range, substantially outperforming all single-layer counterparts. Complementary absorption data for CIP-60/RTV together with a series of multilayered configurations with varied stacking sequences are compiled in Fig. S4. Collectively, these results confirm that the broadband microwave absorption capability is intimately governed by the specific layer arrangement, with each functional layer playing an irreplaceable role in the overall performance.

**Fig. 4:**
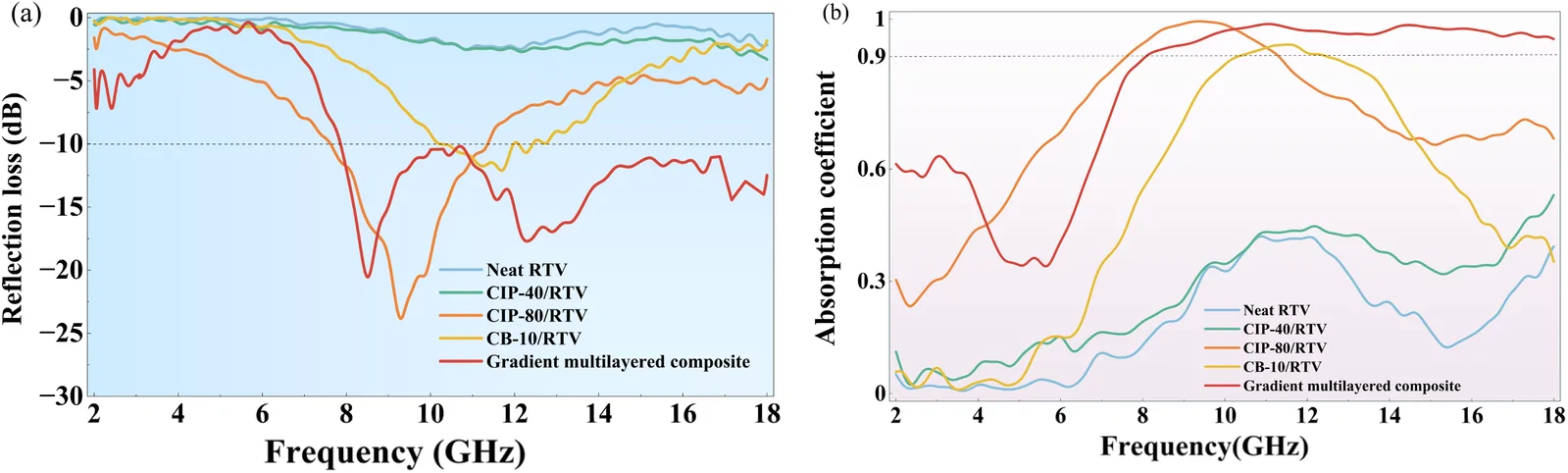
(a) Reflection loss (RL) and (b) corresponding absorption coefficient (*A*) of Neat RTV, CIP-40/RTV, CIP-80/RTV, CB-10/RTV (each 2 mm thick), and the gradient multilayered composite (12 mm total thickness) over 2–18 GHz.

The mechanistic origin of this pronounced broadband absorption is schematically depicted in Fig. 5. The uppermost Neat RTV layer functions as an impedance-matching medium, facilitating microwave entry into the composite with minimal surface reflection. Directly beNeath the Neat RTV layer, the ZnS/RTV layer serves a dual purpose. First, its infrared emissivity can be systematically tuned by varying the ZnS content, thereby enabling precise regulation of thermal radiation for applications such as thermal management and non-contact temperature sensing. Second, it contributes to the gradual impedance transition from free space into the lossy interior by virtue of its relatively lower filler loading compared with the underlying CIP-rich layers.^35^ The gradient CIP/RTV layers that follow, wherein the filler loading increases progressively from top to bottom, constitute the primary attenuation zone where incident electromagnetic energy is gradually dissipated through combined magnetic and dielectric losses. The bottommost CB/RTV layer acts as a built-in reflective substrate, redirecting residual transmitted waves back into the overlying lossy CIP/RTV region for a second attenuation pass while simultaneously promoting destructive interference between incident and reflected waves. This spatial decoupling of wave admission, infrared modulation, progressive dissipation, and internal reflection provides a coherent explanation for the broadband microwave absorption performance observed in Fig. 4.

**Fig. 5:**
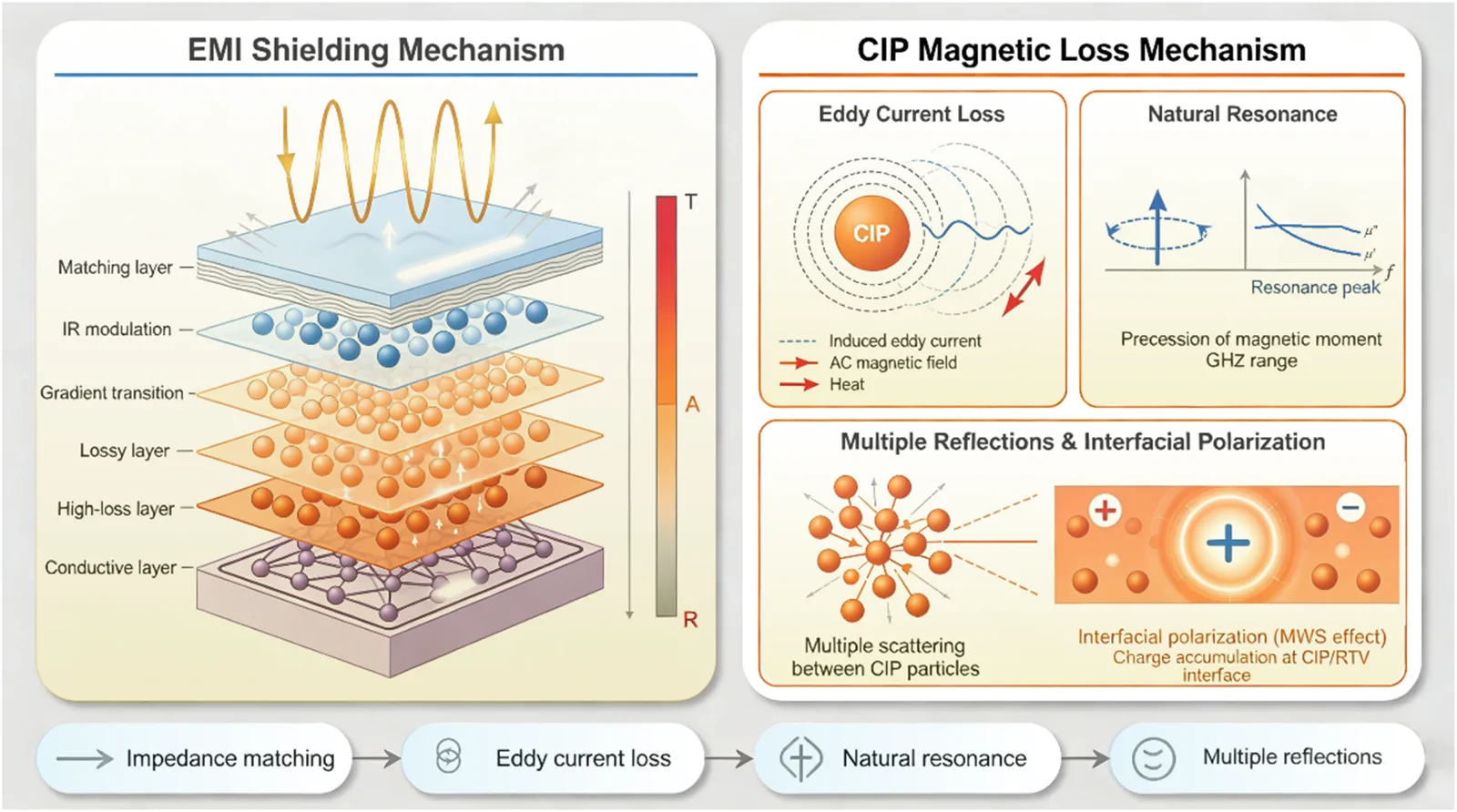
Schematic illustration of the electromagnetic shielding mechanism of the RTV multilayered composite film and the CIP magnetic loss mechanism.

#### 3.2.1 Complex permittivity and permeability of the single layers

To provide a quantitative basis for the subsequent impedance analysis and loss mechanism discussion, the frequency-dependent complex permittivity and permeability of Neat RTV and the CIP-filled single-layer composites were systematically characterized. Fig. 6a–d presents the frequency-dependent complex permittivity (*ε*_r_ = *ε*′ − *jε*″) and complex permeability (*µ*_r_ = *µ*′ − *jµ*″) of Neat RTV and the CIP-filled single-layer composites over 2–18 GHz, obtained from the coaxial transmission line measurements. The real part *ε*′ of all CIP/RTV composites increases progressively with the CIP weight fraction, rising from approximately 3.5 for CIP-40/RTV to approximately 13.7 for CIP-80/RTV (Fig. 6a). The monotonic increase of ε′ with the CIP weight fraction originates from the enhanced interfacial polarization (Maxwell–Wagner–Sillars effect) at the growing density of CIP–RTV interfaces,^36^ as directly evidenced by the homogeneous dispersion of CIP particles in the FESEM images (Fig. 3). Across the measured 2–18 GHz range, *ε*′ remains nearly constant for each composition, indicating the absence of strong dielectric relaxation within this frequency window. The imaginary part *ε*″ (Fig. 6b) remains relatively low for all compositions and exhibits a weak dependence on the CIP content, suggesting that conduction loss and polarization relaxation loss contribute only modestly to the overall dielectric loss.

**Fig. 6:**
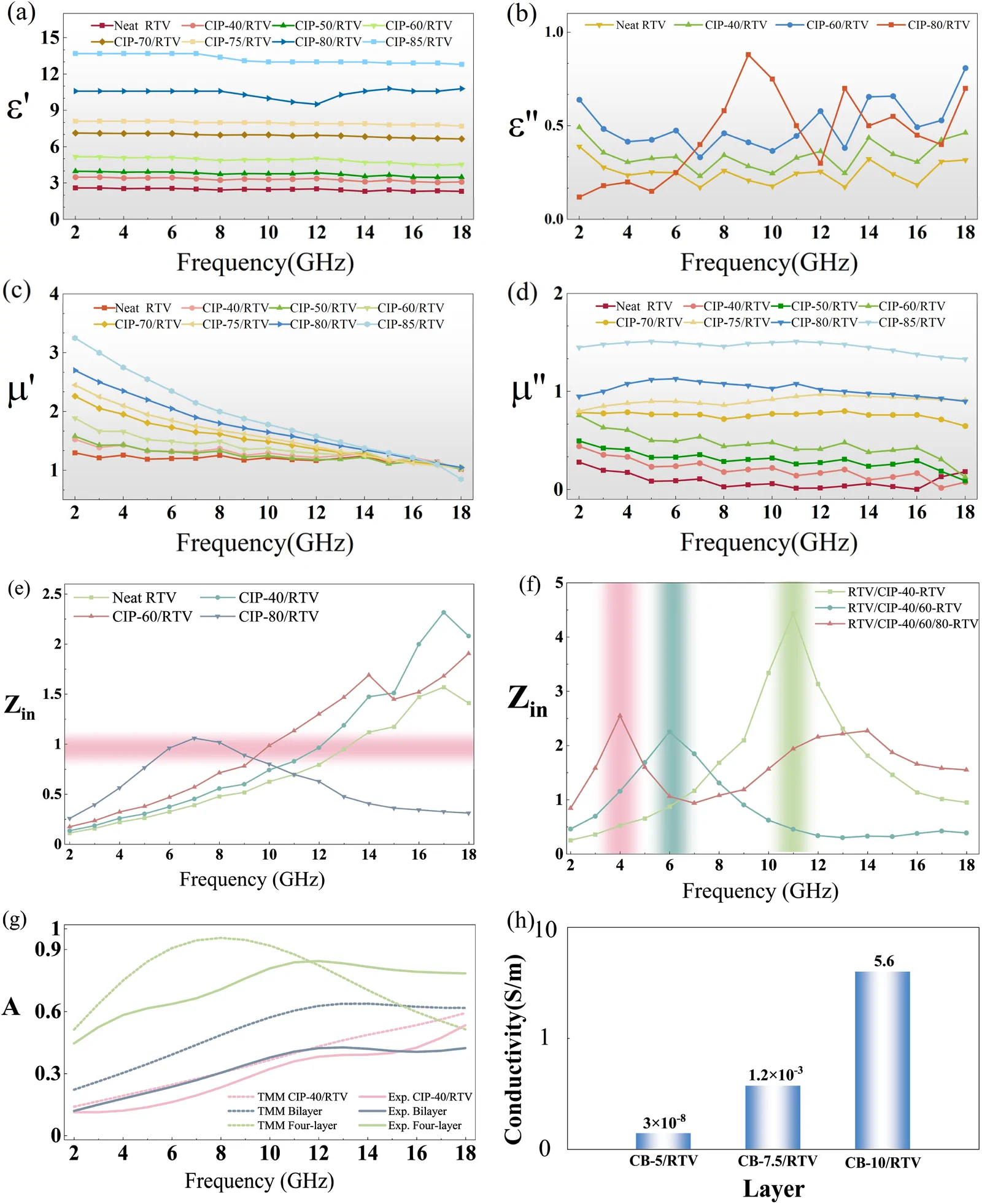
(a–d) Frequency-dependent complex permittivity and permeability of Neat RTV and the single-layer composites. (e, f) Normalized input impedance of the single-layer and multilayered configurations. (g) Cross-validation between the TMM-calculated and arch-method-measured absorption coefficients. (h) DC conductivity of the CB/RTV composites.

The real part *µ*′ and imaginary part *µ*″ of the permeability, shown in Fig. 6c and d, respectively, display a pronounced dependence on the CIP loading. At low frequencies, *µ*′ of CIP-80/RTV reaches a value as high as approximately 3.3, substantially exceeding that of Neat RTV (*µ*′ ≈ 1). All samples exhibit a characteristic frequency dispersion with *µ*′ decreasing monotonically toward higher frequencies, a trend that is most prominent for the highly filled composites. Correspondingly, *µ*″ of the high-loading samples remains at an elevated level over a broad frequency range (2–14 GHz) before declining gradually above 14 GHz, whereas the low-loading samples show considerably weaker magnetic loss.

Collectively, these results establish two key points that underpin the subsequent analysis. First, increasing the CIP loading simultaneously enhances the permittivity and the permeability, yet the former rises far more steeply than the latter, inevitably pushing the characteristic impedance 
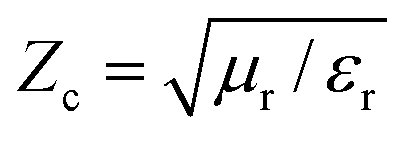
 away from that of free space. Δ*Z* = |*Z*_in_ − 1|, was compared with the measured absorption coefficient *A* for the six-layer gradient composite over the reliable 8–18 GHz range. The Pearson correlation coefficient is *r* = 0.75 indicating a strong positive correlation. Second, the magnetic loss contributed by CIP dominates the overall attenuation capability. To quantitatively evaluate the contribution of each loss mechanism, the dielectric loss tangent (tan *δε* = *ε″*/*ε*′) and magnetic loss tangent (tan *δµ* = *µ″*/*µ*′) were calculated from the measured electromagnetic parameters. For the primary attenuation layer CIP-80/RTV, tan *δµ* is substantially larger than tan *δε* across the entire 2–18 GHz range (*e.g.*, at 10 GHz, tan *δµ* ≈ 0.62 *vs.* tan *δε* ≈ 0.09). For CIP-60/RTV, magnetic loss also dominates, while for CIP-40/RTV, the two mechanisms are comparable. These results quantitatively confirm that magnetic loss is the dominant attenuation mechanism in the gradient composite. The full frequency-dependent curves of tan *δε* and tan *δµ* for all CIP-filled layers are provided in Fig. S7. These two observations provide the experimental foundation for the gradient impedance matching analysis presented in Section 3.2.2 and the discussion of multiple loss mechanisms in Section 3.2.3.

#### 3.2.2 Gradient impedance matching

The broadband absorption behavior observed in Fig. 4 was attributed in the preceding discussion to the gradient impedance matching strategy, wherein the characteristic impedance of the composite is progressively reduced from the top to the bottom layer so that incident microwaves can enter the material with minimal surface reflection. To quantitatively verify this mechanism, the input impedance and theoretical absorption performance of the gradient multilayered composite were calculated using the transmission matrix method (TMM). The frequency-dependent complex permittivity and permeability of each constituent layer, obtained from the coaxial transmission line measurements, served as the sole input parameters. In this framework, the normalized input impedance *Z*_in_ at the front surface of a material directly governs its reflectivity. A *Z*_in_ value approaching unity implies near-perfect impedance matching with free space, allowing incident microwaves to enter with minimal reflection, whereas a value far below unity indicates severe mismatch and strong surface reflection. The intrinsic impedance of free space serves as the reference for this normalization, with 
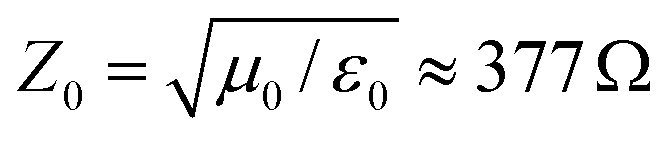
. For a single-layer absorber terminated by a metal backing plate, consistent with the arch method measurement configuration, the normalized input impedance at the front surface is given by eqn (1):^37,38^1
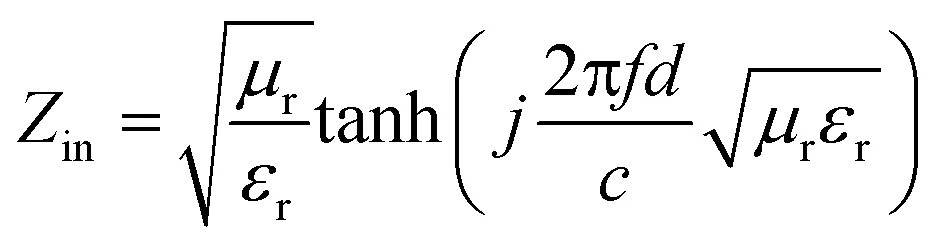
where *d* is the thickness of the layer, *f* is the microwave frequency, and *c* is the speed of light in vacuum. For the gradient multilayered composite, the total input impedance was obtained through a recursive procedure, wherein the output impedance of each layer serves as the load for the layer immediately above it, starting from the short-circuit condition at the metal backing plane. The recursive procedure can be expressed compactly as eqn (2):2
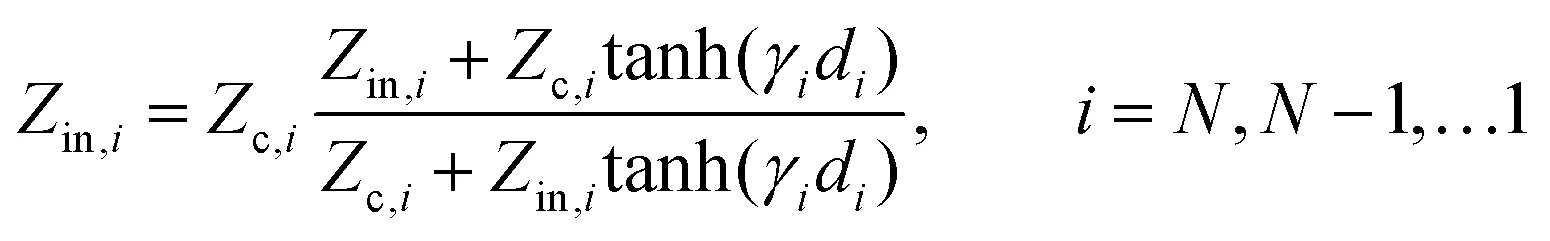
where *Z*_in,*i*_ is the input impedance at the front surface of the *i*-th layer, *Z*_c,*i*_ and *γ*_*i*_ are its characteristic impedance and propagation constant, *d*_*i*_ is its thickness, and *N* is the total number of layers. The recursion is initiated at the bottom layer (*i* = *N*) with the short-circuit condition *Z*_in,*N*+1_ = 0, and terminated at the top layer (*i* = 0), yielding the total input impedance *Z*_in_^total^ = *Z*_in,*i*_*Z*_c_ of the entire multilayered stack. Detailed step-by-step calculations are provided in FS.1.

For Neat RTV, |*Z*_in_| increases monotonically from approximately 0.11 at 2 GHz to 1.57 at 17 GHz, approaching unity only near the upper end of the band (Fig. 6e). This upward trend, characteristic of a low-loss dielectric with increasing electrical thickness, indicates that a favorable impedance matching condition can be achieved at frequencies where the layer thickness approaches an odd multiple of *λ*/4. The intrinsically weak dielectric and magnetic loss of Neat RTV (*ε*″ ≈ 0.1–0.4, *µ*″ ≈ 0.05–0.2), however, precludes any meaningful energy dissipation. Consequently, even at frequencies where |*Z*_in_| approaches unity, the incident microwave energy is not effectively absorbed, consistent with the negligible absorption coefficient (*A* ≈ 0) observed in Fig. 4. For CIP-40/RTV and CIP-60/RTV, the moderate increase in filler content raises both the permittivity and the magnetic loss, yet the resulting |*Z*_in_| remains far from unity across most of the band, with CIP-60/RTV oscillating between 0.14 and 1.91 owing to interference between incident and reflected waves under the metal-backed configuration. At the highest loading, CIP-80/RTV exhibits |*Z*_in_| suppressed below 0.3 over the majority of the 2–18 GHz range, with a broad peak of only ≈1.06 near 7 GHz. This severe impedance mismatch is a direct consequence of the excessively high *ε*′ (up to ≈13.7) induced by the dense CIP loading, which drastically reduces the characteristic impedance 
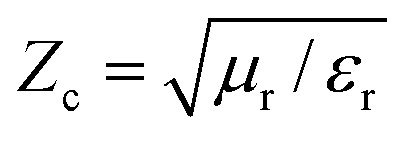
 relative to that of free space. Taken together, these single-layer results illustrate the fundamental dilemma of homogeneous absorbers, in which low filler loadings afford favorable impedance matching but insufficient loss, whereas high filler loadings deliver strong attenuation at the expense of severe impedance mismatch, and neither extreme can satisfy the dual requirements for broadband microwave absorption.

Fig. 6f displays the |*Z*_in_| of three multilayered configurations, RTV/CIP-40-RTV (bilayer, 4 mm), RTV/CIP-40/60-RTV (three-layer, 6 mm), and RTV/CIP-40/60/80-RTV (four-layer, 8 mm). The bilayer structure exhibits a pronounced resonance peak with |*Z*_in_| reaching ≈4.4 at 11 GHz, followed by a sharp decline toward higher frequencies. This intense oscillation, arising from the abrupt impedance discontinuity between only two distinct layers, confines the favorable matching condition (|*Z*_in_| close to unity) to a narrow frequency interval near 16–17 GHz. The three-layer configuration partially suppresses this resonance, with the peak amplitude reduced to ≈2.25 and shifted downward to ≈6 GHz, reflecting the damping effect of the additional CIP-60 layer. The four-layer structure further attenuates the oscillatory behavior, yielding a markedly flattened |*Z*_in_| profile that remains within 1.0–1.2 over the 4–8 GHz range. This emerging plateau signifies a qualitative transition from narrow-band resonant matching to broadband impedance matching, achieved through the progressive smoothing of the impedance gradient. Notably, the peak amplitude decreases from ∼4.4 (bilayer) to ∼2.25 (three-layer) to ∼2.55 (four-layer), while the peak frequency shifts downward, directly demonstrating that the introduction of additional transitional layers progressively suppresses the interference-driven resonances that plague simple bilayer designs. A direct comparison of Fig. 6e and f reveals a clear evolutionary trend, in which the input impedance curve transforms from a sharp, narrow-band resonant profile toward a flattened, broadband matched profile as the number of impedance-graded layers increases.

The reliability of the TMM approach is further assessed by comparing the calculated absorption coefficients with the corresponding arch-method measurements (Fig. 6g). For the single-layer and bilayer configurations, the calculated and measured curves exhibit satisfactory agreement across the entire frequency band, with deviations generally confined within ±0.15. This consistency validates the accuracy of the measured electromagnetic parameters and the reliability of the recursive TMM formalism for structures with a limited number of well-defined interfaces. For the four-layer configuration, the agreement remains favorable at higher frequencies (9–18 GHz), while a discernible discrepancy emerges in the 5–8 GHz region, where the TMM overestimates the absorption coefficient by approximately 0.4–0.7.

This deviation may be attributed to the combined effects of interfacial imperfections and slight thickness variations across the internal interfaces, which are not accounted for in the idealized TMM model. Previous studies on multilayer absorbers have demonstrated that abundant heterogeneous interfaces introduce additional interfacial polarization and weak reflections, which can locally alter the impedance matching condition.^39,40^ Moreover, thickness deviations in multilayered architectures are known to shift the effective electrical thickness and thus the resonance frequency, as reviewed in the context of multi-layered electromagnetic screening designs.^53^ Despite this localized deviation, the overall trend predicted by TMM agrees well with the experimental results, confirming the validity of the gradient impedance matching mechanism.

#### 3.2.3 Full-wave electromagnetic simulation

The impedance analysis in Section 3.2.2 establishes that the gradient design enables microwaves to enter the composite with minimal reflection. To visualize the subsequent attenuation, full-wave simulations were conducted using CST Microwave Studio. The model was validated against the measured absorption coefficients of the gradient composite, CIP-40/RTV, and CIP-80/RTV. As shown in Fig. 7a, the simulated and experimental curves exhibit close agreement over the entire 2–18 GHz range, with RMSE values remaining below 0.09 for all three configurations, confirming the accuracy of the assigned electromagnetic parameters.

**Fig. 7:**
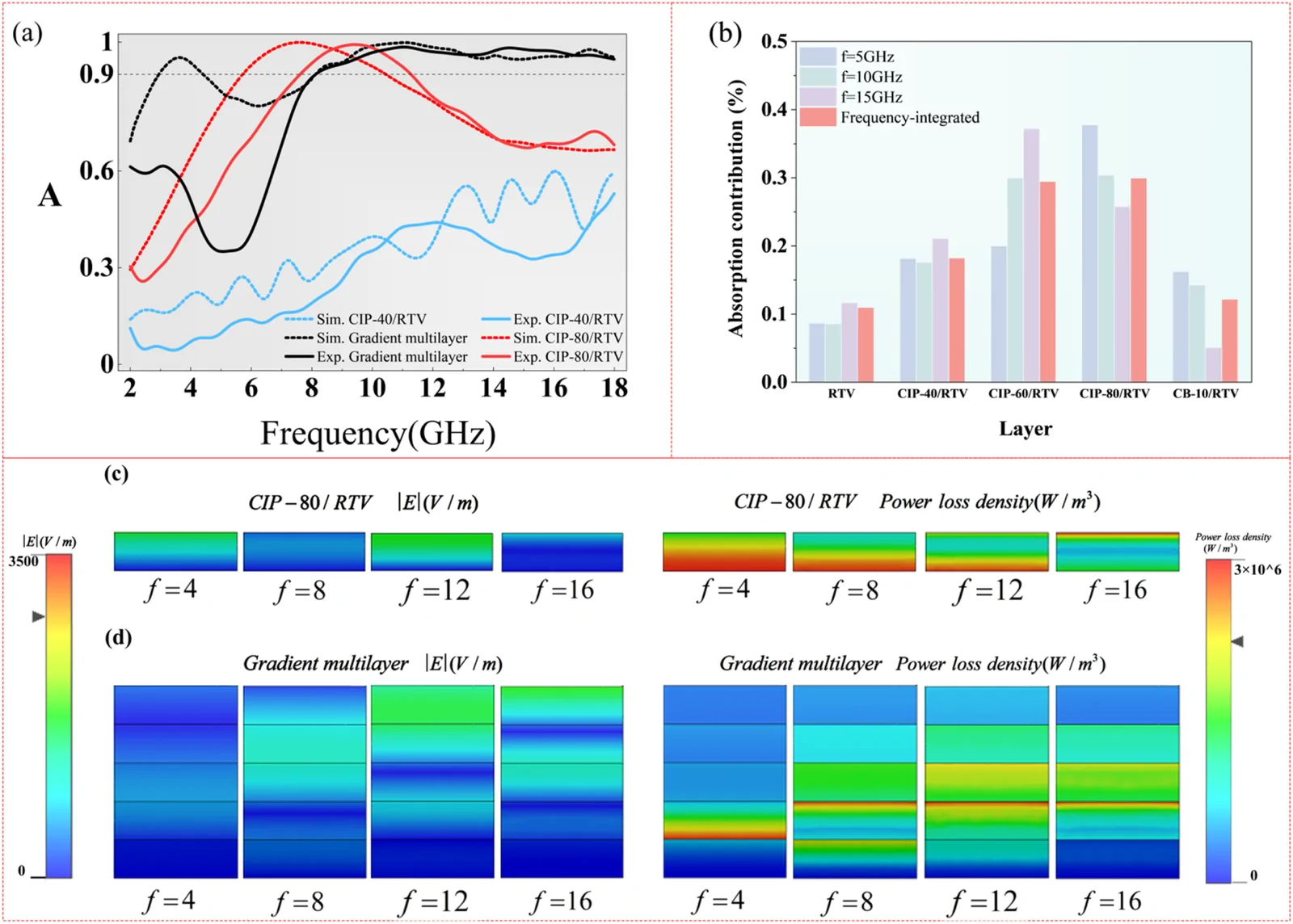
Full-wave electromagnetic simulation results. (a) Simulated *vs.* measured absorption coefficients. (b) Layer-resolved power absorption fraction. (c) Electric field and power loss distributions of CIP-80/RTV. (d) Electric field and power loss distributions of the gradient multilayered composite.

Fig. 7c and d present the electric field and power loss distributions for CIP-80/RTV and the gradient composite at 4, 8, 12, and 16 GHz. In CIP-80/RTV, a pronounced standing-wave pattern appears near the front surface across all frequencies, with alternating bright and dark fringes signaling severe impedance mismatch that traps incident energy in a surface oscillation. The corresponding power loss distribution reinforces this picture. At 4 GHz the dissipation concentrates near the bottom of the layer, yet as the frequency increases to 12 and 16 GHz the dominant loss zone migrates upward, eventually localizing near the top surface. This upward shift indicates that high-frequency microwaves are increasingly reflected or absorbed before they can penetrate into the deeper region of the homogeneous layer.

The gradient composite displays a fundamentally different behavior. The electric field decays smoothly and progressively from the top to the bottom layers at all frequencies, with the field intensity transitioning gradually from green and light-blue in the upper layers to blue in the lower layers, and no standing-wave features are observed anywhere in the stack. This smooth attenuation profile directly visualizes the consequence of the gradient impedance matching strategy. The power loss distribution further reveals where this attenuation takes place. At 4 GHz a broad high-loss zone appears in the CIP-80 layer, appearing as a distinct red band. As the frequency increases to 12 and 16 GHz, this loss band narrows and shifts toward the interfaces of the CIP-80 layer, consistent with the reduced penetration depth imposed by the skin effect.^54^ Critically, the dissipation remains confined within the CIP-60, CIP-80, and CB-10 layers across the entire frequency range and never drifts toward the incident layer.

Fig. 7b quantifies the absorption contribution of each layer, integrated over the full 2–18 GHz band and evaluated at 5, 10, and 15 GHz. CIP-60 and CIP-80 together account for approximately 60% of the total dissipation, whereas the top RTV layer contributes roughly 11%, consistent with its designated matching function. The absorption fraction of CIP-60 rises with frequency while that of CIP-80 declines, driven by the frequency-dependent penetration depth. This redistribution demonstrates that the gradient architecture confines energy dissipation within the interior lossy layers across the entire band, providing direct evidence that the spatial decoupling of wave admission and energy dissipation constitutes the physical origin of the broadband absorption performance.

#### 3.2.4 Role of the conductive CB layer

The gradient impedance matching and loss distribution analyses presented in the preceding sections establish that the CIP-filled layers serve as the primary attenuation zones. The bottom CB layer, however, performs a fundamentally distinct function that is essential to the overall absorption performance.

The DC conductivity of the CB/RTV composites, measured by the four-point probe method, exhibits a pronounced percolation behavior (Fig. 6h). CB-5/RTV registers a low conductivity of 3 × 10^−8^ S m^−1^, indicating that the CB aggregates remain isolated below the percolation threshold. At 7.5 wt% loading, the conductivity rises to 1.2 × 10^−3^ S m^−1^, signaling the onset of a connected conductive network. Further increasing the CB content to 10 wt% elevates the conductivity to 5.6 S m^−1^. This value exceeds the benchmark of approximately 1 S m^−1^, above which a material is generally regarded as an efficient microwave reflector owing to its drastically reduced skin depth and high surface reflectivity. The CB-10/RTV layer therefore possesses sufficient conductivity to function as an effective reflective boundary within the gradient stack.

To evaluate the specific contribution of the CB layer, two control experiments were performed. First, a five-layer composite without the CB bottom layer (Neat/ZnS-20/CIP-40/60/80-RTV) was prepared and measured by the arch method under the same metal-backed condition as the six-layer composite. As shown in Fig. 8a, the absorption coefficients of the six-layer and five-layer composites are close across the entire 2–18 GHz range. This is because the metal backing plate already provides a nearly perfect reflective boundary, and the CB layer, possessing comparable reflectivity, does not introduce significant additional reflection under this condition. The two reflective boundaries are functionally overlapping, and the CB layer does not markedly alter the overall absorption when a metal backing is present.

**Fig. 8:**
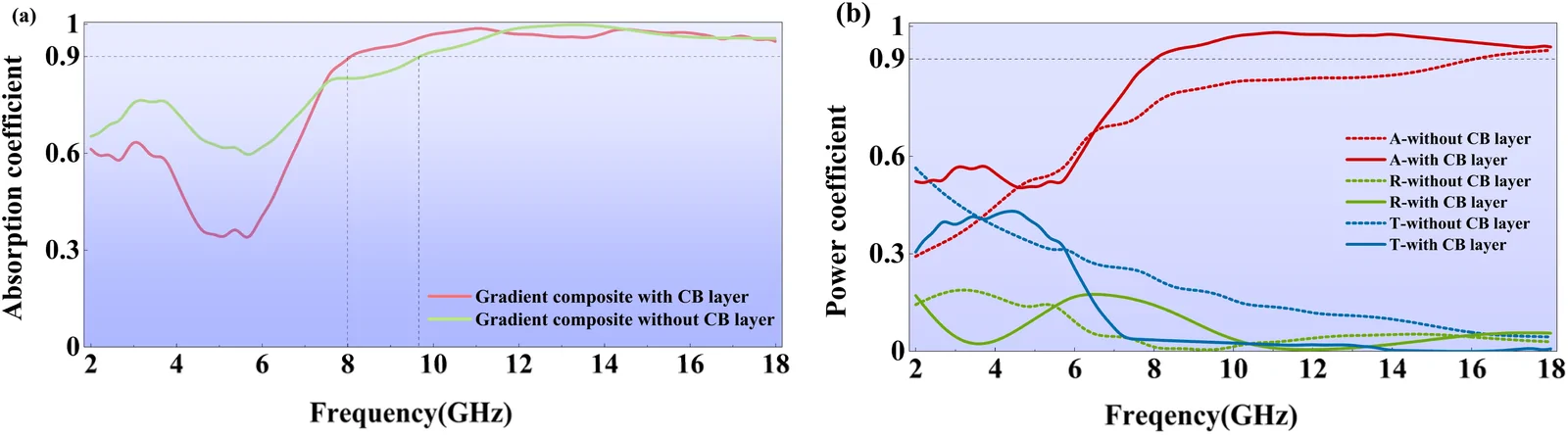
Role of the CB bottom layer in the gradient multilayered composite. (a) Absorption coefficients of the composite with and without the CB bottom layer measured by the arch method under metal-backed conditions over 2–18 GHz. (b) Simulated power coefficients (*A*, *R*, *T*) of the composite with and without the CB bottom layer under free-space conditions over 2–18 GHz.

Second, CST simulations were performed under free-space conditions (without a metal backing plate) to examine whether the CB layer can function as an internal reflective boundary in practical scenarios where an external metal backing is absent. Fig. 8b presents the simulated absorption (*A*), reflection (*R*), and transmission (*T*) coefficients of the six-layer composite with and without the CB layer. In the presence of the CB layer, the transmission coefficient remains low and the absorption coefficient remains high across the entire band, approaching the performance of the metal-backed case. Without the CB layer, however, the transmission coefficient increases significantly and the absorption coefficient drops markedly, confirming that the CB layer is essential for maintaining absorption performance in the absence of an external metal backing. These results demonstrate that the CB layer acts as a built-in reflective substrate, redirecting residual microwaves back into the overlying lossy CIP layers for a second attenuation pass and promoting destructive interference between incident and reflected waves.^55^

The contrast between the behavior of CB-10/RTV in isolation and as an integral component of the gradient architecture further underscores the importance of structural design. As shown in Fig. 4, single-layer CB-10/RTV delivers only a moderate absorption coefficient, whereas the same material, when positioned beneath the gradient CIP layers, contributes decisively to the broadband absorption performance. The conductive CB layer is therefore not employed as an independent absorber but rather as a functional reflector whose properties are fully exploited through rational integration into the multilayered architecture. This design principle transforms a highly conductive layer from a passive reflector into an active contributor to absorption, complementing the gradient impedance matching strategy and completing the spatial decoupling of wave admission, attenuation, and reflection.

#### 3.2.5 Comparison with previously reported absorbers

To benchmark the broadband microwave absorption performance of the gradient multilayered composite against the current state of the art, Fig. 9 presents a comparison of the effective absorption bandwidth (*A* > 0.9, equivalent to RL < −10 dB) and minimum reflection loss with representative polymer-based microwave absorbers reported in recent years.^41–52^ The rectangle height represents the sample thickness. The gradient multilayered composite achieves an effective absorption bandwidth of 10 GHz (8–18 GHz), which compares favorably with the best values reported for flexible polymer-based absorbers. Detailed information on the selected references, including material composition, filler type, RLmin, EAB, and thickness, is provided in Table S3.

**Fig. 9:**
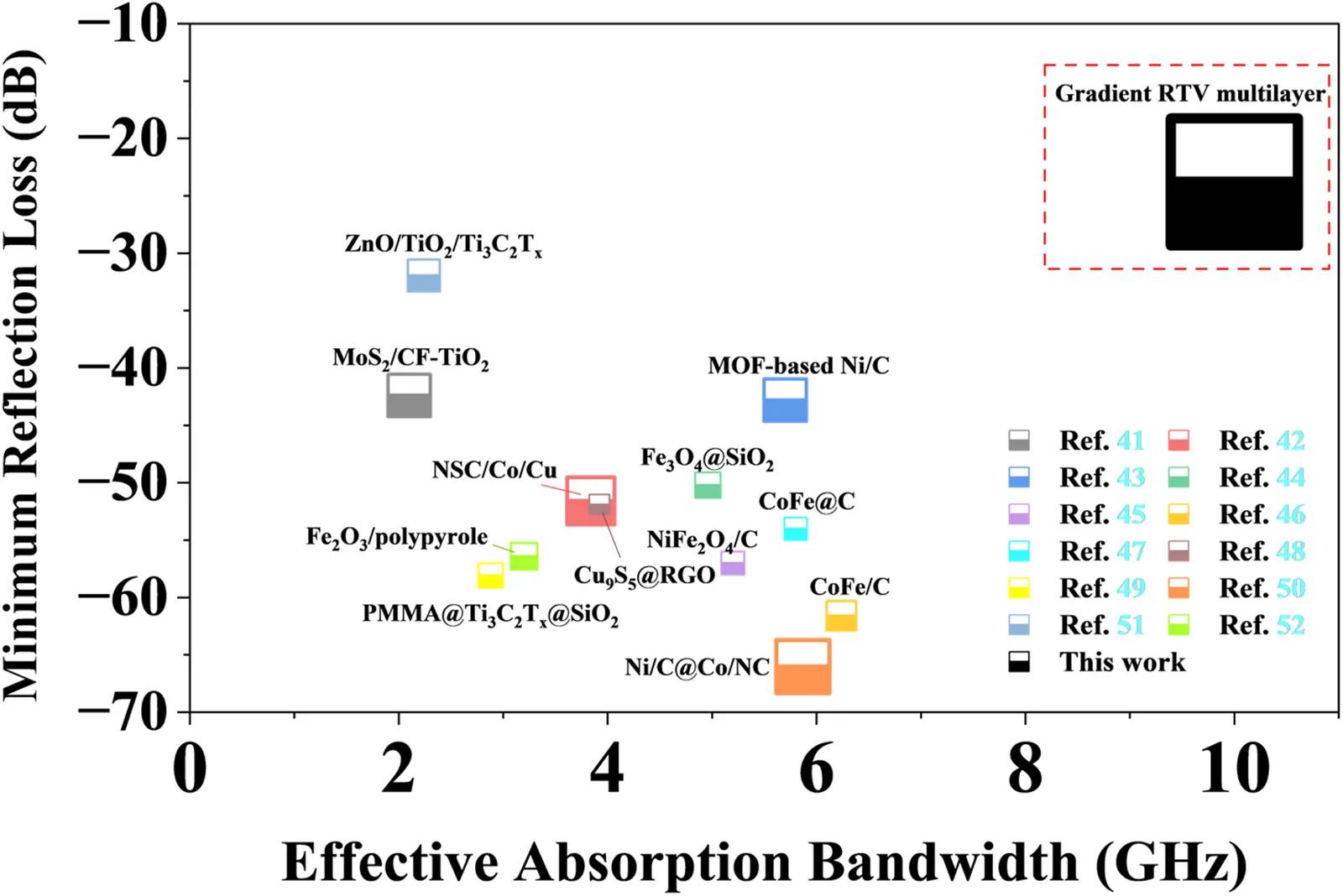
Comparison of the gradient multilayered composite with recently reported microwave absorbers in terms of effective absorption bandwidth (EAB) and minimum reflection loss (RLmin). The rectangle height represents the sample thickness.

As shown in Fig. 9, most reported absorbers exhibit effective absorption bandwidths below 6 GHz, regardless of their minimum reflection loss. Several materials achieve exceptionally low RLmin values (below −50 dB), yet these deep absorption peaks are typically confined to narrow frequency ranges. In contrast, the gradient multilayered composite occupies a distinct position at the far right of the comparison, reflecting its broadband absorption capability that is not compromised by the pursuit of an extremely low single-frequency reflection minimum. This performance profile is a direct consequence of the gradient impedance matching strategy, which maintains favorable impedance matching across a wide frequency range rather than optimizing absorption at a single resonant frequency.

It should be noted that absorption bandwidth is strongly thickness-dependent. The present composite has a total thickness of 12 mm, which is larger than some thin-film absorbers. This thickness is a deliberate design choice to accommodate the six-layer gradient architecture that simultaneously provides broadband absorption, infrared modulation, and mechanical flexibility. Therefore, the comparison should be interpreted with thickness in mind. The combination of a 10 GHz effective absorption bandwidth, mechanical flexibility, and infrared modulation capability distinguishes the present work from the majority of existing absorbers, even when thickness is taken into account.

### 3.3. Infrared modulation performance

The preceding sections have demonstrated that the gradient multilayered architecture delivers broadband microwave absorption through the synergistic action of impedance matching, progressive attenuation, and conductive reflection. Beyond this electromagnetic function, the composite was designed to simultaneously provide infrared modulation capability through the incorporation of a ZnS/RTV layer. This section evaluates the infrared performance of the relevant single-layer composites that constitute the functional building blocks of the gradient stack.

Fig. 10b presents the surface emissivity of Neat RTV and the single-layer composites measured in the 3–5 µm and 8–12 µm atmospheric windows at room temperature. Neat RTV exhibits a high emissivity of approximately 0.93–0.95 in both bands (Table S2), consistent with the intrinsically strong infrared radiation of silicone elastomers. CIP-80/RTV and CB-10/RTV display similarly high emissivity values, indicating that the magnetic and conductive fillers contribute negligibly to infrared modulation. In contrast, the incorporation of ZnS leads to a systematic reduction in surface emissivity. As the ZnS content increases from 10 wt% to 30 wt%, the emissivity in the 3–5 µm band decreases progressively, demonstrating that the infrared emissivity of the composite can be effectively tuned by varying the ZnS loading. This tunability originates from the intrinsically low emissivity of ZnS in the infrared atmospheric window, which suppresses the overall thermal radiation of the composite.^56^

**Fig. 10:**
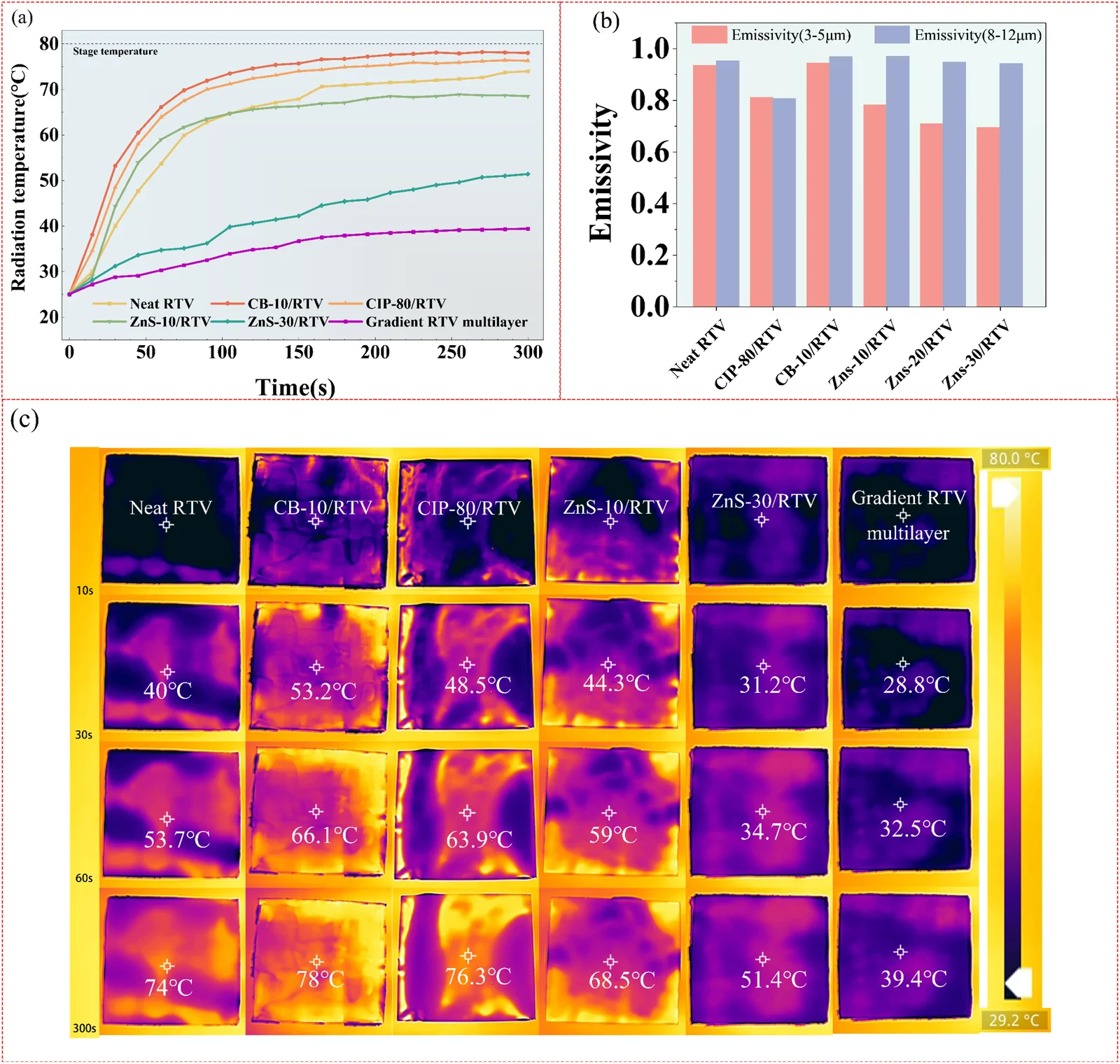
Infrared modulation performance of the RTV-based composites. (a) Radiation temperature as a function of heating time on an 80 °C heating stage. (b) Surface emissivity of the single-layer composites in the 3–5 µm and 8–12 µm atmospheric windows. (c) Time-lapse infrared thermal images of the RTV-based composites.

To evaluate the dynamic infrared modulation capability, Neat RTV, CIP-80/RTV, CB-10/RTV, ZnS-10/RTV, ZnS-30/RTV and Gradient RTV multilayer (Neat/ZnS-20/CIP-40/60/80/CB-10-RTV) were placed on an 80 °C heating stage and their radiation temperatures were monitored over time. Fig. 10a displays the corresponding temperature profiles. All samples start from a similar initial temperature. As heating progresses, the radiation temperatures of all samples rise rapidly during the first 20 s before gradually approaching their respective steady-state values. Neat RTV, CIP-80/RTV, and CB-10/RTV reach the highest steady-state temperatures, clustering closely together. ZnS-10/RTV, although exhibiting a similarly rapid initial temperature rise, ultimately stabilizes at a temperature approximately 5.5 °C lower than that of Neat RTV. ZnS-30/RTV displays the lowest steady-state radiation temperature, with a total reduction of approximately 22.6 °C relative to Neat RTV. This progressive suppression of the steady-state radiation temperature with increasing ZnS content directly reflects the corresponding reduction in surface emissivity observed in Fig. 10b. A lower emissivity surface radiates less thermal energy at a given physical temperature, thereby reducing the apparent temperature registered by the infrared camera.

The gradient multilayered composite exhibits a temperature profile that differs from both neat RTV and the single-layer ZnS/RTV samples. Its steady-state radiation temperature is approximately 34.6 °C lower than that of neat RTV. The surface emissivity of the gradient composite is approximately 0.945 in the 8–12 µm band, which is close to that of neat RTV (0.953). This is because the top neat RTV layer is optically thick in the thermal infrared band, and its intrinsic emissivity dominates the surface radiative properties of the multilayer stack. The lower radiation temperature of the gradient composite is therefore attributed primarily to the thermal barrier effect of the gradient structure, in which the high-filler inner layers reduce the effective thermal conductivity and delay heat transfer from the heated stage to the emitting surface. This mechanism is distinct from the emissivity-driven modulation observed in the single-layer ZnS/RTV samples, where the low-emissivity ZnS filler directly suppresses surface thermal radiation. The gradient architecture thus provides effective infrared modulation through a combination of surface emissivity control and thermal management, with the latter becoming dominant in the complete multilayer configuration.

Fig. 10c provides the time-lapse infrared thermal images of Neat RTV, CIP-80/RTV, CB-10/RTV, ZnS-30/RTV and Gradient RTV multilayer (Neat/ZnS-20/CIP-40/60/80/CB-10-RTV) recorded at 10, 30, 60 and 300 s. At *t* = 10 s, ZnS-30/RTV already appears noticeably darker than the other three samples, a direct consequence of its suppressed thermal radiation. By 30 s, neat RTV, CIP-80/RTV, and CB-10/RTV have brightened considerably and stand out prominently in the images, whereas ZnS-30/RTV remains substantially darker. At 60 s, the visual contrast between ZnS-30/RTV and the other three samples becomes even more pronounced. The gradient composite also appears darker than neat RTV at all time points, consistent with its lower steady-state radiation temperature. These observations are fully consistent with the temperature profiles in Fig. 10a and confirm that the ZnS layer effectively suppresses infrared radiation through its reduced surface emissivity, while the gradient architecture provides additional thermal management through its layered structure.

Taken together, the results in this section confirm that the ZnS/RTV layer functions as an effective infrared modulation medium, and that its integration into the gradient architecture endows the composite with tunable infrared emissivity and reduced radiation temperature. The gradient design enables the composite to simultaneously deliver broadband microwave absorption and infrared modulation, demonstrating the multifunctional advantage made possible by assigning distinct optical and electromagnetic functions to spatially separated layers.

### 3.4. Mechanical tensile properties

The broadband microwave absorption and infrared modulation capabilities of the gradient multilayered composite have been demonstrated in the foregoing sections. For practical deployment in flexible and conformable applications, the material must also withstand handling and bending without catastrophic failure. This section examines the tensile properties of the constituent single-layer materials and the full gradient composite, with particular attention to the correlation between the mechanical behavior and the morphological characteristics revealed by FESEM.

Fig. 11 presents representative tensile stress–strain curves of Neat RTV, CIP-40/RTV, CIP-70/RTV, CIP-80/RTV, unmodified CIP-70/RTV, CB-10/RTV, ZnS-20/RTV, and the gradient multilayered composite. The corresponding tensile strength, elongation at break, elastic modulus, and toughness are summarized in Table 2.

**Fig. 11:**
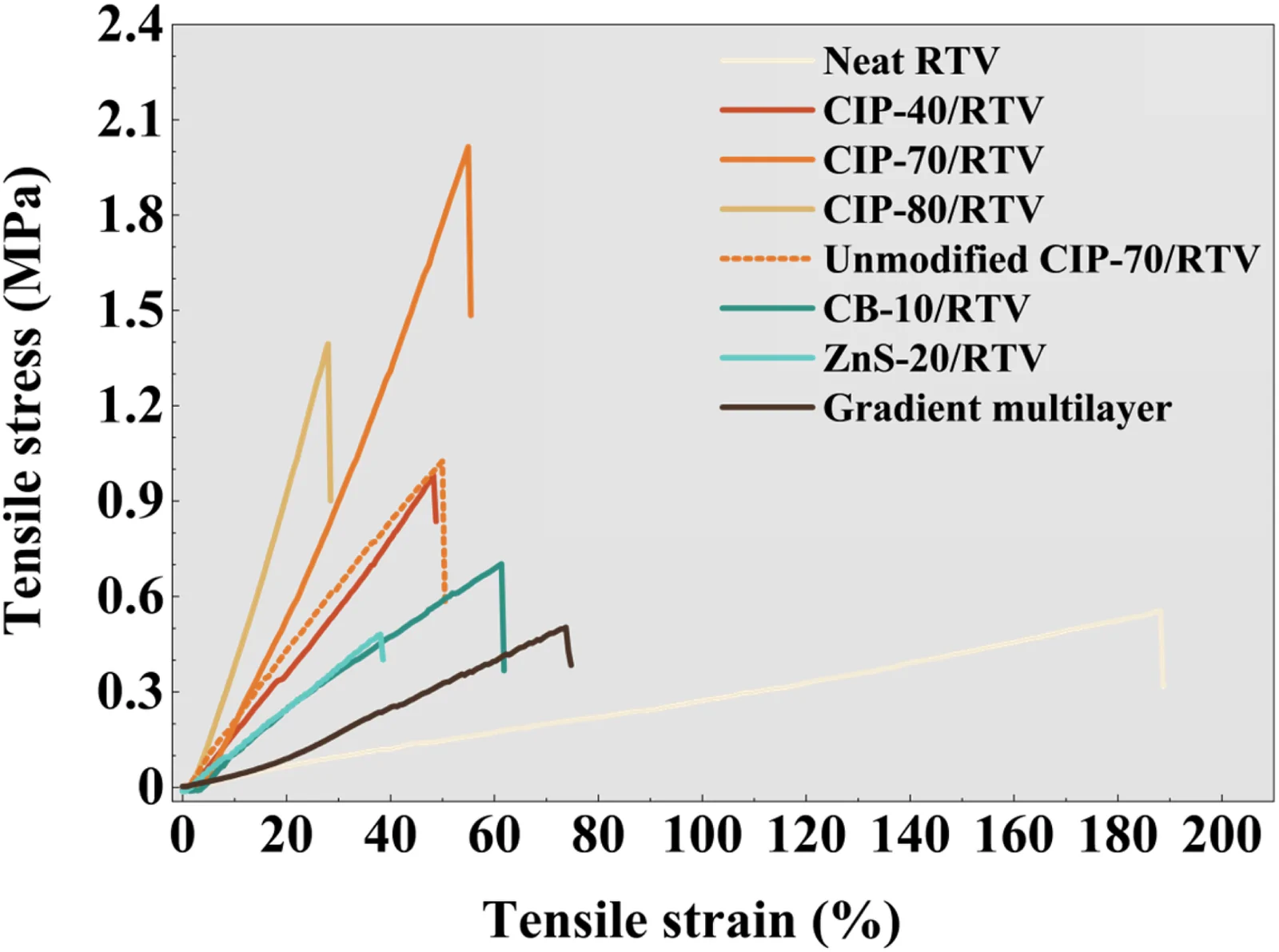
Tensile stress–strain curves of Neat RTV, CIP-40/RTV, CIP-70/RTV, CIP-80/RTV, unmodified CIP-70/RTV, CB-10/RTV, ZnS-20/RTV, and the gradient multilayered composite.

**Table 2:** Tensile properties of Neat RTV and the RTV-based single-layer and gradient multilayered composites^a^

Sample	Tensile strength (MPa)	Elongation at break (%)	Elastic modulus (MPa)	Toughness (MJ m^−3^)
Neat RTV	0.55 ± 0.03	188.3 ± 12.5	0.36 ± 0.02	0.52 ± 0.04
CIP-40/RTV	0.98 ± 0.05	48.3 ± 3.2	1.10 ± 0.08	0.31 ± 0.02
CIP-70/RTV	2.01 ± 0.12	56.2 ± 4.1	0.79 ± 0.06	0.50 ± 0.05
CIP-80/RTV	1.39 ± 0.10	28.5 ± 2.9	1.73 ± 0.15	0.19 ± 0.02
Unmodified CIP-70/RTV	1.02 ± 0.08	50.9 ± 3.8	1.64 ± 0.11	0.25 ± 0.03
CB-10/RTV	0.70 ± 0.04	61.4 ± 4.5	0.84 ± 0.07	0.21 ± 0.02
ZnS-20/RTV	0.47 ± 0.09	38.1 ± 3.7	0.13 ± 0.04	0.12 ± 0.02
Gradient multilayer	0.57 ± 0.10	73.8 ± 4.2	0.37 ± 0.09	0.21 ± 0.04

a (Data are presented as mean ± standard deviation (SD) from at least three parallel specimens).

Neat RTV exhibits a tensile strength of 0.55 MPa and an elongation at break of 188.3%, characteristic of a highly flexible, unfilled silicone elastomer. Upon incorporation of CIP, the tensile strength initially rises, reaching a maximum of 2.01 MPa at 70 wt%, before declining to 1.39 MPa at 80 wt%. This non-monotonic trend is directly correlated with the morphological evolution observed in the FESEM images (Fig. 3). At 70 wt% loading, the modified CIP particles are uniformly dispersed within the RTV matrix with strong interfacial adhesion, which facilitates efficient stress transfer and delays the onset of fracture. At 80 wt%, however, the FESEM images reveal a densely packed filler network with partial agglomeration and reduced interparticle spacing, which introduces additional stress concentration sites and leads to premature failure. The elongation at break decreases monotonically with increasing CIP content, from 188.3% to 28.5%, reflecting the progressive restriction of polymer chain mobility by the rigid filler network.

A direct comparison between CIP-70/RTV and unmodified CIP-70/RTV highlights the efficacy of KH-570 surface modification. The modified sample achieves nearly twice the tensile strength (2.01 MPa *versus* 1.02 MPa) and retains a higher elongation at break (56.2% *versus* 50.9%). This improvement is directly supported by the FESEM observations (Fig. 3). The unmodified composite exhibits severe filler agglomeration, with large CIP clusters distributed throughout the matrix. These agglomerates act as stress concentration sites during tensile loading, initiating premature fracture and limiting the ultimate strength. In contrast, the modified composite displays a homogeneous dispersion of CIP particles with intimate contact between the filler and the RTV matrix, enabling effective stress transfer and delaying the onset of failure.

The toughness, calculated as the area under the stress–strain curve up to break, is also summarized in Table 2. CIP-70/RTV exhibits the highest toughness among the CIP-filled composites (0.50 MJ m^−3^), owing to its combination of high tensile strength and moderate elongation. In contrast, CIP-80/RTV shows a significantly lower toughness (0.19 MJ m^−3^), as the high filler loading severely restricts ductility. The unmodified CIP-70/RTV displays a lower toughness ([*X*] MJ m^−3^) than its modified counterpart, consistent with its premature failure caused by agglomeration. Neat RTV, despite its low strength, achieves a high toughness (0.52 MJ m^−3^) due to its exceptional elongation at break.

The CB-filled and ZnS-filled composites were evaluated for completeness. CB-10/RTV exhibits a tensile strength of 0.70 MPa and an elongation at break of 61.4%, while ZnS-20/RTV displays a lower strength of 0.47 MPa and a limited elongation of 38.1%. Their relatively low toughness values (0.21 and 0.12 MJ m^−3^, respectively) are attributed to the rigid filler networks formed by the chain-like CB aggregates and the weakly bonded ZnS particles, as evidenced by the FESEM images. Their primary functions within the gradient architecture are conductive reflection and infrared modulation, respectively, rather than structural load bearing.

The gradient multilayered composite exhibits a tensile strength of 0.57 MPa, an elongation at break of 73.8%, an elastic modulus of 0.37 MPa, and a toughness of 0.21 MJ m^−3^. Its strength and modulus are nearly identical to those of Neat RTV, indicating that the ductile outer layers dominate the mechanical response. This structural integrity enables the ductile outer layers (Neat RTV and ZnS/RTV) to accommodate the majority of the tensile deformation while protecting the brittle inner layers (CIP-80 and CB-10) from premature failure. As a result, the elongation at break of the gradient composite is approximately 2.6 times that of CIP-80/RTV and 1.9 times that of ZnS-20/RTV. Its toughness is higher than that of the brittle inner layers (CIP-80/RTV and ZnS-20/RTV) but lower than Neat RTV and CIP-70/RTV, indicating a favorable balance between flexibility and multifunctional performance. This mechanical profile confirms that the gradient architecture preserves structural integrity under deformation without compromising the broadband microwave absorption and infrared modulation capabilities.

## 4. Conclusions

In summary, a gradient multilayered room-temperature-vulcanized silicone rubber composite was successfully constructed through a sequential layer-by-layer casting strategy. By spatially distributing carbonyl iron powder, conductive carbon black, and zinc sulfide across distinct layers with progressively increasing filler content, the competing requirements of impedance matching, electromagnetic attenuation, conductive reflection, and infrared modulation were effectively decoupled within a single flexible architecture.

The gradient multilayered composite achieves a broadband absorption coefficient A exceeding 0.9 across 8–18 GHz. This broadband microwave absorption performance substantially surpasses that of all single-layer counterparts and arises from the gradient impedance matching mechanism. The top Neat RTV layer, possessing a low permittivity, serves as an impedance-matching entrance that allows incident microwaves to penetrate with minimal surface reflection. The underlying CIP/RTV layers with gradient filler loadings then progressively attenuate the propagating wave through combined magnetic and dielectric losses, while the highly conductive CB/RTV bottom layer functions as a built-in reflective substrate that redirects residual transmitted waves back into the lossy region for a second attenuation pass. This spatial decoupling of wave admission, progressive dissipation, and internal reflection was quantitatively verified through transmission matrix method calculations and directly visualized by full-wave electromagnetic simulations of electric field and power loss density distributions.

The incorporation of a ZnS/RTV layer imparts tunable infrared emissivity to the composite. The surface emissivity decreases systematically with increasing ZnS content, and dynamic thermal imaging on an 80 °C heating stage confirms that ZnS-30/RTV achieves a steady-state radiation temperature reduction of approximately 22.6 °C relative to Neat RTV. This infrared modulation capability is achieved without compromising the broadband microwave absorption performance. Mechanically, the gradient multilayered composite retains a tensile strength of 0.50 MPa and an elongation at break of 73.8%, which is approximately 2.6 times that of the individual brittle functional layers. The elastic modulus remains nearly identical to that of Neat RTV, indicating that the ductile outer layers dominate the mechanical response and protect the inner high-filler layers from premature failure. The KH-570 surface modification of CIP particles significantly enhances the tensile strength and ductility of the attenuation layers through improved interfacial adhesion and filler dispersion.

This work demonstrates that a rationally designed gradient multilayered architecture can reconcile the inherent trade-offs among broadband microwave absorption, infrared modulation, and mechanical flexibility that have long constrained homogeneous composites. The structure–property relationships established herein offer a versatile design platform for flexible multifunctional composites applicable to wearable electronics, thermal management, and multispectral compatibility scenarios. Nevertheless, systematic durability tests such as cyclic bending and thermal aging were not performed in this study, and will be the subject of future work to fully assess the service life of the composite under practical service conditions.

## Author contributions

Li Hao: conceptualization investigation, writing –original draft. Zhao Shuwei: methodology. Tu Qunzhang: funding acquisition. Shen Xinmin: supervision. Peng Wenqiang: software, funding acquisition. Liu Qing: validation, formal analysis. Yin Qin: writing – review editing. Ma Yao: visualization.

## Conflicts of interest

The authors declare no conflict of interest.

## Supplementary Material

RA-016-D6RA07751J-s001

## Data Availability

Data will be made available on request. Supplementary information (SI): digital photographs, additional absorption coefficient data for CIP/RTV composites at intermediate loadings, detailed transmission matrix method calculations, CST simulation parameters, surface emissivity measurements, and extra tensile stress–strain curves for various single-layer composites. See DOI: https://doi.org/10.1039/d6ra07751j.
