# Supplementary material for: Gradient multilayered RTV silicone rubber composites with carbonyl iron, conductive carbon, and ZnS for broadband microwave absorption and infrared stealth
Source: RSC Adv. 2026 Oct 8;16(46):60855–71. doi: 10.1039/d6ra07751j (PMC13646912; doi:10.1039/d6ra07751j)
Supplement: Supplementary file 1 [file RA-016-D6RA07751J-s001.pdf]

Supplementary Information (SI) for RSC Advances.
This journal is © The Royal Society of Chemistry 2026

Supporting Information

Gradient Multilayered RTV Silicone Rubber Composites with Carbonyl Iron, Conductive Carbon, and ZnS for Broadband Microwave Absorption and Infrared Stealth

Li Hao[a], Zhao Shuwei[a], Tu Qunzhang[a], Shen Xinmin[a,*],Peng Wenqiang[b], Liu Qing[a], Yin Qin[a], Ma Yao[a]

[a]*Army Engineering University, Nanjing, Jiangsu, 210007, China*

[b]*National University of Defense Technology, Changsha, Hunan,410073, China*

Email:

Li hao: lhazcy@outlook.com

Shen Xinmin: xmshen2014@aeu.edu.cn

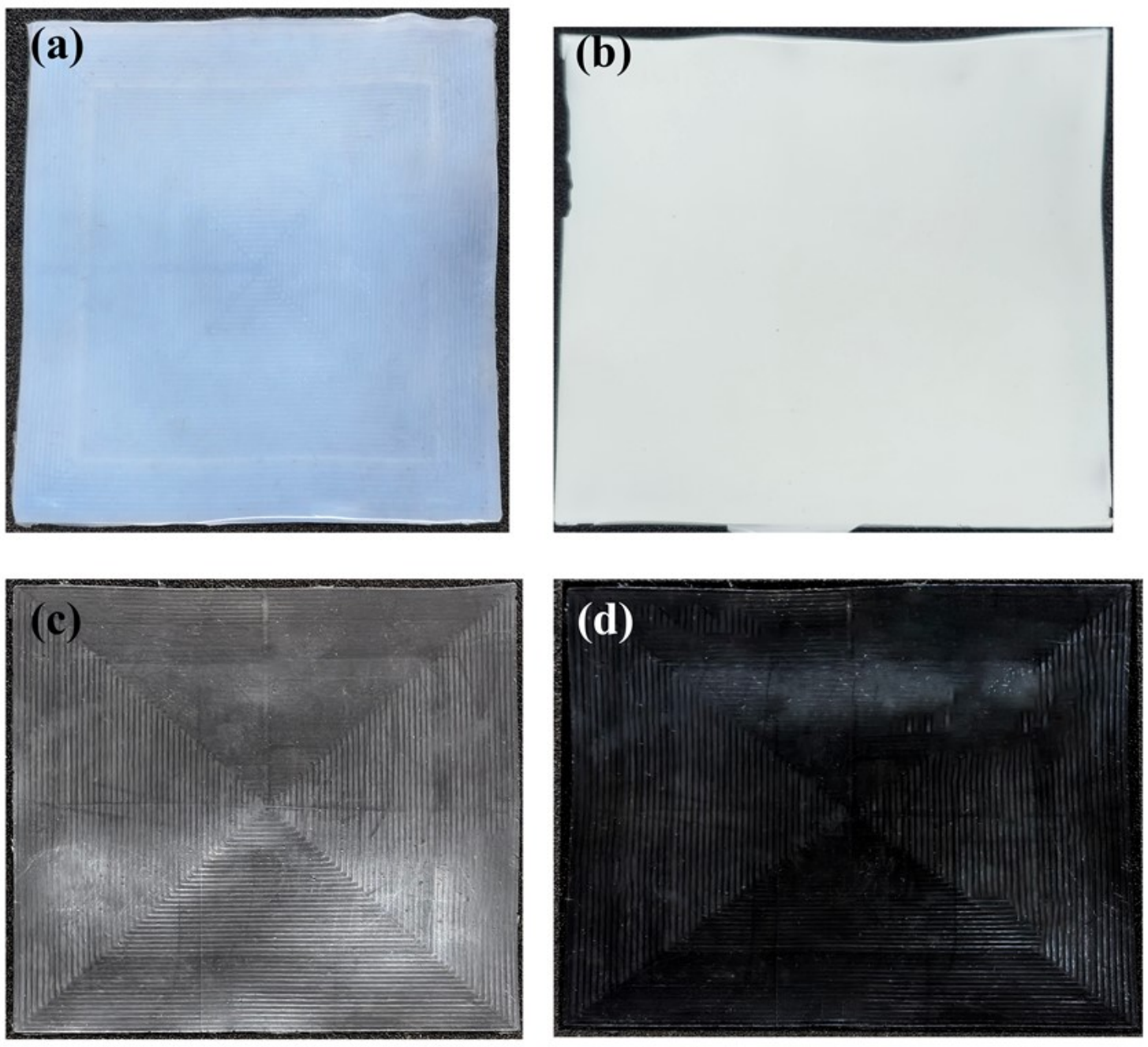

Fig. S1. Digital photographs of neat RTV and the representative single-layer RTV-based composites: (a) Neat RTV, (b) ZnS/RTV, (c) CIP/RTV, and (d) CB/RTV.

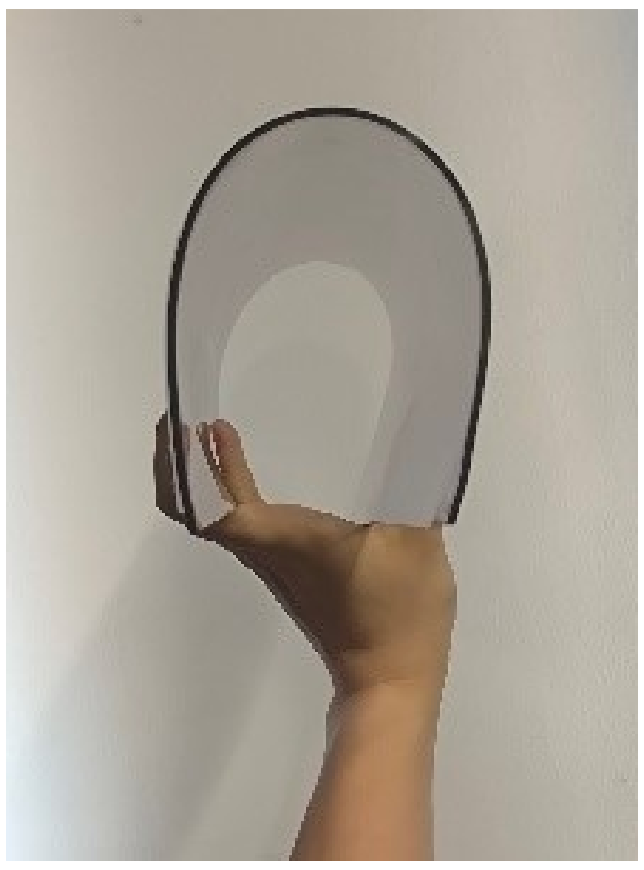

Fig. S2. Digital photographs demonstrating the excellent mechanical flexibility of the gradient multilayered composite, highlighting its structural integrity for conformal applications without compromising the multifunctional performance.

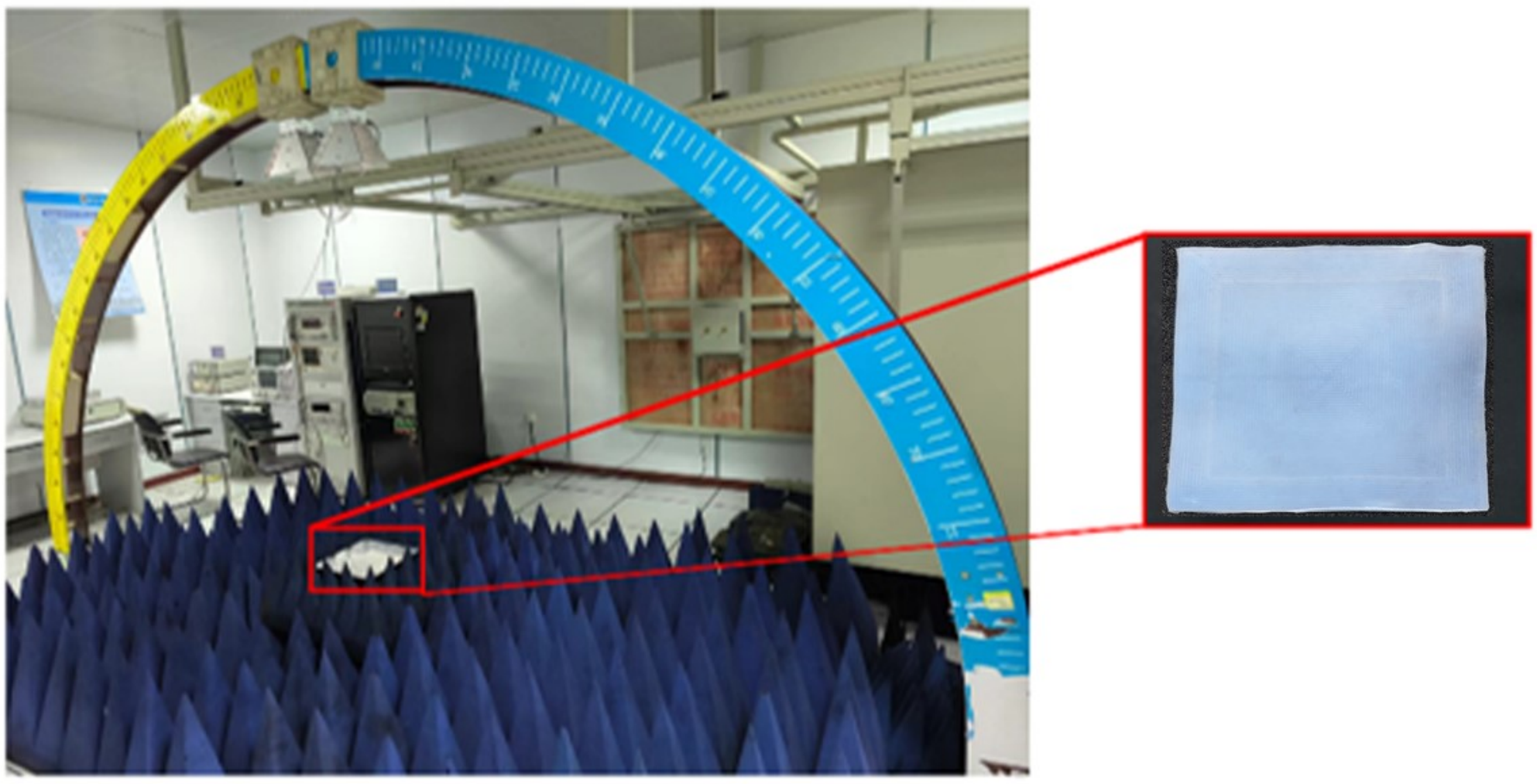

Fig. S3. Digital photograph of the arch method measurement setup used for evaluating the reflection loss of the composites, showing the vector network analyzer, the arched frame with.

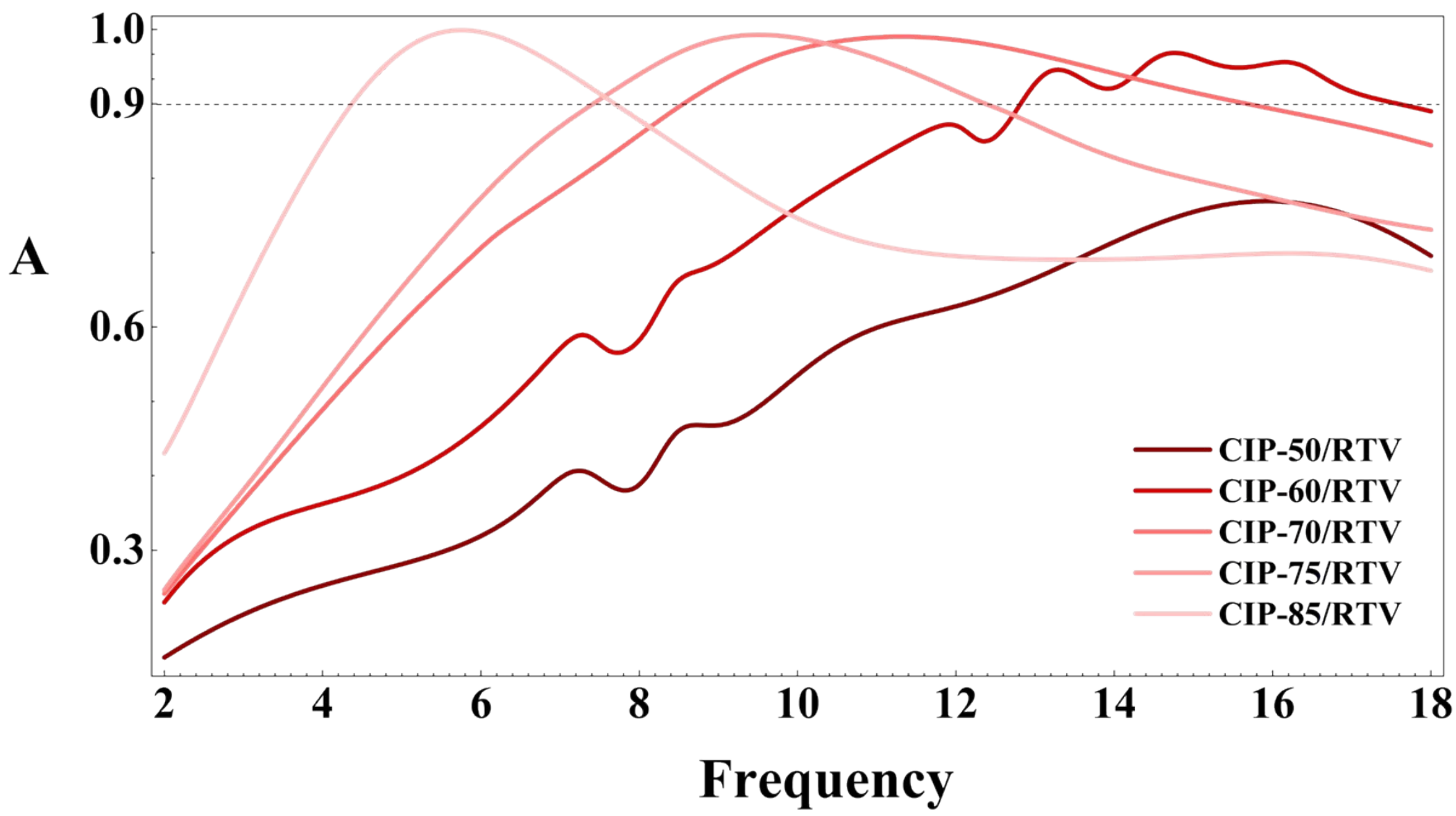

Fig. S4. Frequency-dependent absorption coefficient (A) of CIP/RTV composites with intermediate CIP loadings (50, 60, 70, 75, and 85 wt%) over 2–18 GHz.

S1. Detailed Transmission Matrix Method (TMM) Calculation

Tab. S1. Electromagnetic parameters of each layer at 10 GHz.

Layer	ε'	ε''	μ'	μ''
RTV (Layer 1)	2.46	0.18	1.22	0.06
CIP-40/RTV (Layer 2)	3.31	0.25	1.30	0.22
CIP-60/RTV (Layer 3)	4.95	0.36	1.37	0.47
CIP-80/RTV (Layer 4)	9.75	0.89	1.65	1.03

All layers have the same thickness $d = 2$ mm. The speed of light $c = 3 \times 10^8$ m/s.

Step 1: Calculate the Characteristic Impedance and Propagation Constant of Each Layer

For each layer i, the complex permittivity and permeability are:

$$\varepsilon_{r,i} = \varepsilon_i' - j\varepsilon_i'', \mu_{r,i} = \mu_i' - j\mu_i''$$

The characteristic impedance $Z_{c,i}$ (normalized to free space) and the propagation constant γ_i are calculated as:

$$Z_{c,i} = \sqrt{\frac{\mu_{r,i}}{\varepsilon_{r,i}}}, \gamma_i = j\frac{2\pi f}{c}\sqrt{\mu_{r,i}\varepsilon_{r,i}}$$

At 10 GHz Layer 1 (RTV):

$$\varepsilon_{r,1} = 2.46 - j0.18, \mu_{r,1} = 1.22 - j0.06$$

$$Z_{c,1} = \sqrt{\frac{1.22 - j0.06}{2.46 - j0.18}} \approx 0.704 - j0.006$$

$$\gamma_1 d = j\frac{2\pi \times 10^{10} \times 0.002}{3 \times 10^8}\sqrt{(1.22 - j0.06)(2.46 - j0.18)} \approx 0.023 + j0.727$$

Layer 2 (CIP-40/RTV):

$$\varepsilon_{r,2} = 3.31 - j0.25, \mu_{r,2} = 1.30 - j0.22$$

$$Z_{c,2} = \sqrt{\frac{1.30 - j0.22}{3.31 - j0.25}} \approx 0.629 - j0.020$$

$$\gamma_2 d = j\frac{2\pi \times 10^{10} \times 0.002}{3 \times 10^8}\sqrt{(1.30 - j0.22)(3.31 - j0.25)} \approx 0.047 + j0.865$$

Layer 3 (CIP-60/RTV):

$$\varepsilon_{r,3} = 4.95 - j0.36, \mu_{r,3} = 1.37 - j0.47$$

$$Z_{c,3} = \sqrt{\frac{1.37 - j0.47}{4.95 - j0.36}} \approx 0.531 - j0.037$$

$$\gamma_3 d = j\frac{2\pi \times 10^{10} \times 0.002}{3 \times 10^8}\sqrt{(1.37 - j0.47)(4.95 - j0.36)} \approx 0.071 + j1.088$$

Layer 4 (CIP-80/RTV):

$$\varepsilon_{r,4} = 9.75 - j0.89, \mu_{r,4} = 1.65 - j1.03$$

$$Z_{c,4} = \sqrt{\frac{1.65 - j1.03}{9.75 - j0.89}} \approx 0.416 - j0.057$$

$$\gamma_4 d = j\frac{2\pi \times 10^{10} \times 0.002}{3 \times 10^8}\sqrt{(1.65 - j1.03)(9.75 - j0.89)} \approx 0.147 + j1.690$$

Step 2: Recursive Calculation of the Input Impedance

The recursive formula for the input impedance is:

$$Z_{\text{in},i} = Z_{c,i}\frac{Z_{\text{in},i+1} + Z_{c,i}tanh(\gamma_i d)}{Z_{c,i} + Z_{\text{in},i+1}tanh(\gamma_i d)}$$

The recursion starts from the bottom layer (Layer 4) with the short-circuit termination at the metal backing plane ($Z_{\text{in},5} = 0$).

Layer 4 (CIP-80/RTV):

$$Z_{\text{in},4} = Z_{c,4}\tanh(\gamma_4 d) \approx 0.387 + j0.161$$

$|Z_{\text{in},4}| \approx 0.419$

Layer 3 (CIP-60/RTV, load = $Z_{\text{in},4}$):

$$Z_{\text{in},3} = Z_{c,3}\frac{Z_{\text{in},4} + Z_{c,3}tanh(\gamma_3 d)}{Z_{c,3} + Z_{\text{in},4}tanh(\gamma_3 d)} \approx 0.614 + j0.414$$

$$|Z_{\text{in},3}| \approx 0.740$$

Layer 2 (CIP-40/RTV, load = $Z_{\text{in},3}$):

$$Z_{\text{in},2} = Z_{c,2}\frac{Z_{\text{in},3} + Z_{c,2}tanh(\gamma_2 d)}{Z_{c,2} + Z_{\text{in},3}tanh(\gamma_2 d)} \approx 1.221 + j1.137$$

$$|Z_{\text{in},2}| \approx 1.668$$

Layer 1 (RTV, load = $Z_{\text{in},2}$):

$$Z_{\text{in},1} = Z_{c,1}\frac{Z_{\text{in},2} + Z_{c,1}tanh(\gamma_1 d)}{Z_{c,1} + Z_{\text{in},2}tanh(\gamma_1 d)} \approx 1.219 + j0.619$$

$|Z_{\text{in},1}| \approx 1.367$

Step 3: Calculate the Reflection and Absorption Coefficients

The voltage reflection coefficient Γ and the power absorption coefficient A are:

$$\Gamma = \frac{Z_{\text{in},1} - 1}{Z_{\text{in},1} + 1}, A = 1 - |\Gamma|^2$$

At 10 GHz:

$\Gamma \approx 0.118 + j0.223, |\Gamma| \approx 0.252$

$A \approx 1 - 0.064 \approx 0.936$

Summary

At 10 GHz, the four-layer RTV/CIP-40/CIP-60/CIP-80-RTV composite achieves an absorption coefficient of approximately **0.94**, confirming that over 90% of the incident microwave power is dissipated within the gradient structure. This numerical example illustrates the recursive TMM procedure used throughout Section 3.2.2 for calculating the input impedance and absorption performance of arbitrary multilayered configurations.

S2. CST Simulation Details

Full-wave electromagnetic simulations were performed using CST Microwave Studio (Frequency Domain Solver) to visualize the electric field distribution and volumetric power loss density within the multilayered composites. The simulation setup is detailed below.

Geometry and Material Assignment

A three-dimensional multilayer model was constructed with the same layer sequence and thicknesses as the experimental gradient composite. Each layer was modeled as a 2 mm thick slab with in-plane dimensions of 18 mm × 18 mm, and assigned the measured frequency-dependent complex permittivity and permeability obtained from the coaxial transmission line measurements. The material properties were imported into the CST material library as frequency-dependent data points and interpolated over the 2–18 GHz range.

Boundary Conditions

A perfect electric conductor (PEC) boundary was applied to the bottom surface of the model to replicate the metal backing plate used in the arch method measurement. Perfectly matched layer (PML) boundaries were applied to the top and bottom open surfaces to absorb outgoing waves without reflection. Periodic boundary conditions were imposed on the lateral sides to emulate an infinite planar multilayer structure.

Excitation

A linearly polarized plane wave with an electric field amplitude of 1 V/m was normally incident on the top surface of the multilayer stack. The incident wave propagated along the stacking direction, consistent with the configuration of the arch method measurement.

Mesh Settings

A tetrahedral mesh with adaptive refinement was employed. The maximum mesh element size was set to $\lambda/10$ at the highest simulation frequency (18 GHz), where λ is the wavelength within each respective material layer. This ensured sufficient spatial resolution for accurate field and loss density calculations.

Solver Configuration

The frequency-domain solver was used to compute the electromagnetic field distributions at discrete frequencies of 4, 8, 12, and 16 GHz. The S-parameters were recorded at each frequency and post-processed to obtain the absorption coefficient for comparison with the experimental data.

Post-Processing

The electric field magnitude $|E|$ and volumetric power loss density distributions were extracted from the simulation results on the central cross-sectional plane of the model. For quantitative analysis of the layer-resolved absorption, the volume loss in each individual layer was integrated over the entire layer volume and normalized to the total power dissipated across all layers.

Tab. S2. Surface emissivity of the RTV-based composites measured in the 3–5 μm and 8–12 μm atmospheric windows at room temperature.

Sample	Emissivity (3–5 μm)	Emissivity (8-12 μm)
Neat RTV	0.935	0.953
CIP-40/RTV	0.863	0.881
CIP-60/RTV	0.83	0.836
CIP-80/RTV	0.811	0.807
CB-10/RTV	0.944	0.969
Zns-10/RTV	0.781	0.971
Zns-15/RTV	0.741	0.958
Zns-20/RTV	0.708	0.947
Zns-25/RTV	0.705	0.939
Zns-30/RTV	0.695	0.942
Gradient RTV multilayer	0.933	0.945

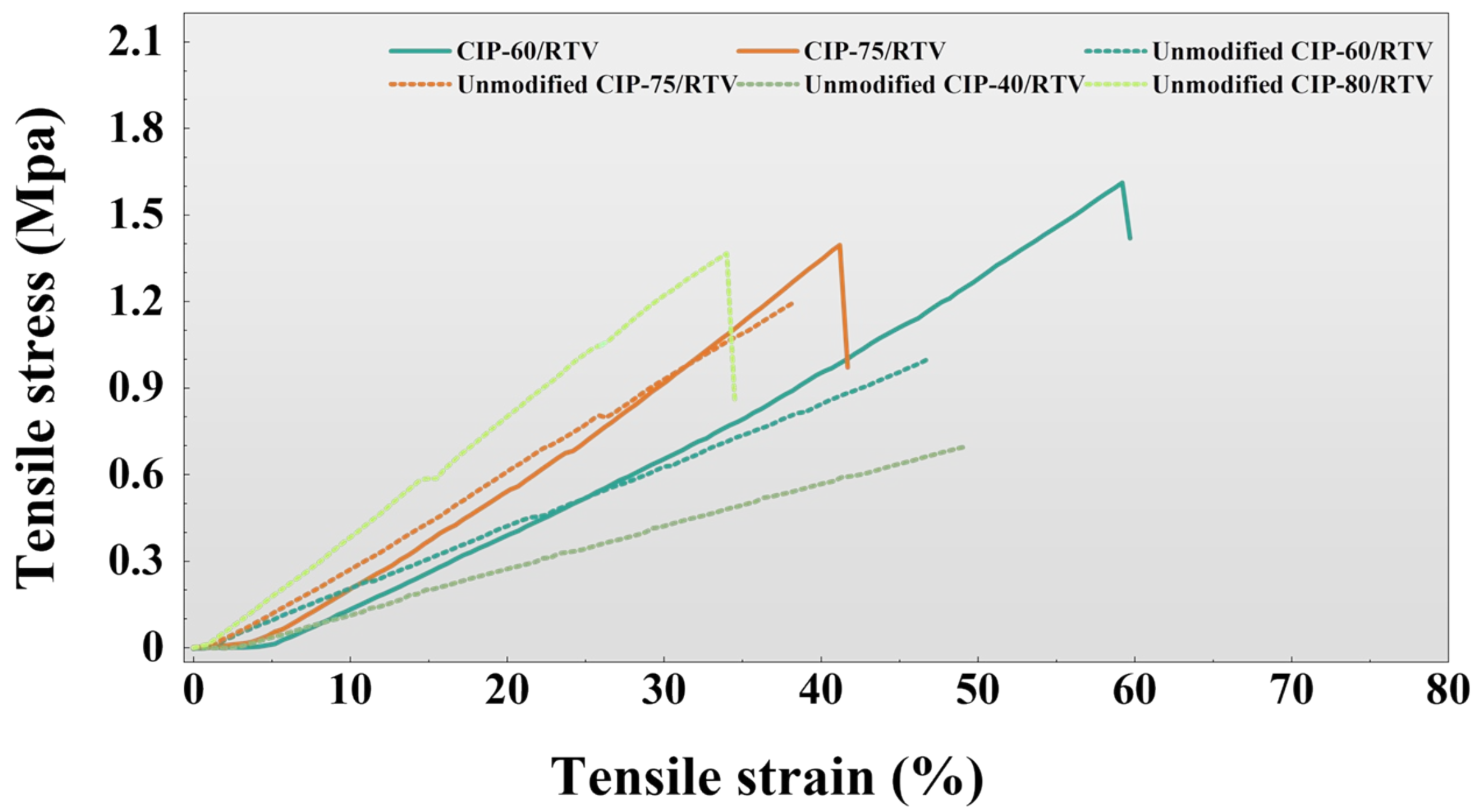

Fig. S5. Representative tensile stress–strain curves of unmodified CIP/RTV composites and selected modified CIP/RTV composites with intermediate filler loadings, illustrating the effect of KH-570 surface treatment on the mechanical behavior of CIP-filled RTV systems.

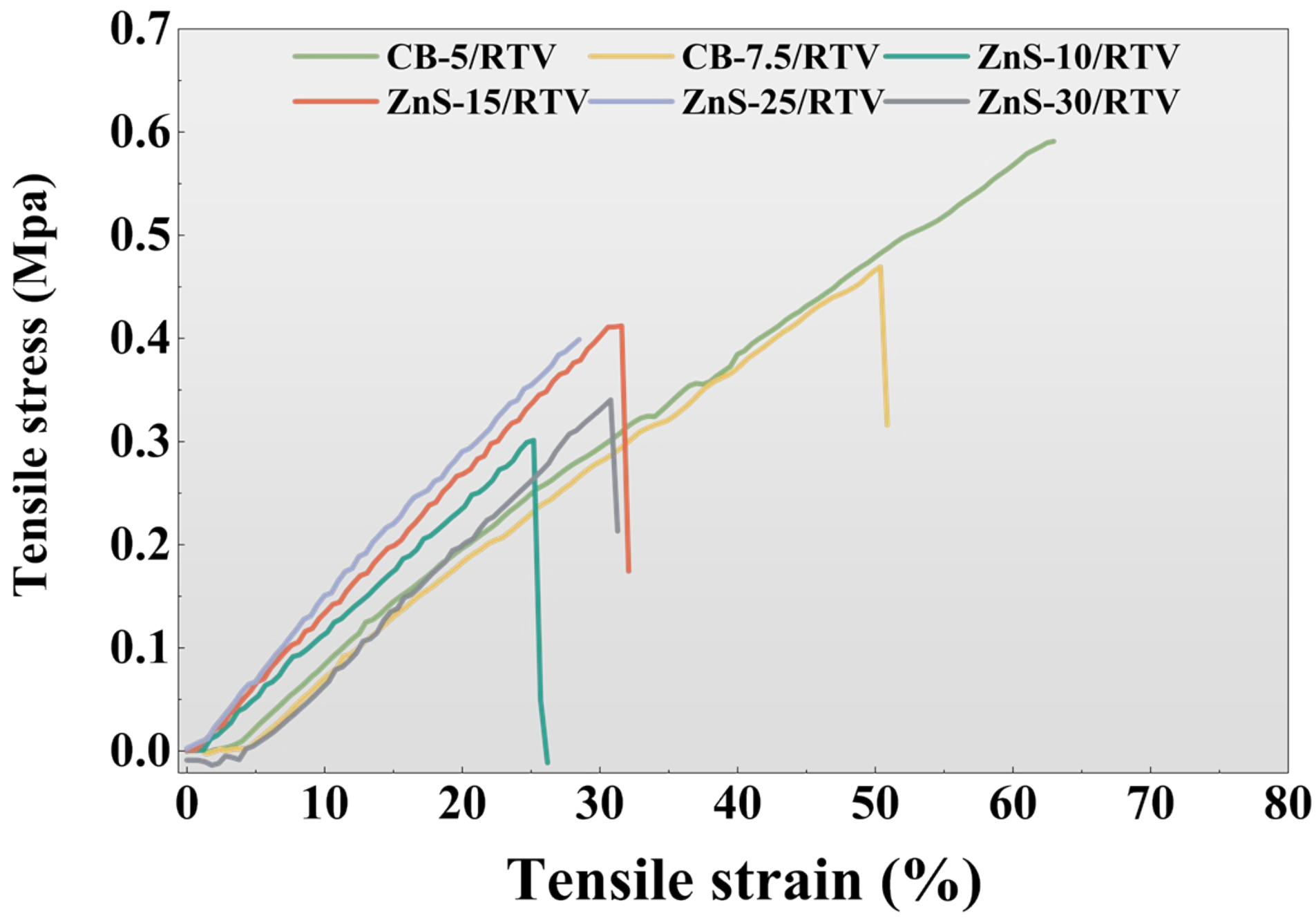

Fig. S6. Representative tensile stress–strain curves of CB-filled and ZnS-filled single-layer RTV composites, showing the mechanical response of the conductive reflection and infrared modulation functional layers.

Tab. S3. Comparison of microwave absorption performance between the gradient multilayered RTV composite in this work and representative absorbers reported in recent literature.

Number	Materials	Frequency band (GHz)	EAB (GHz)	RLmin (dB)	Ref.	Year	Journal	Thickness (mm)
1	Gradient RTV multilayer	8.0-18.0	10.0	-23.8	This work	-	-	12
2	MoS_2/CF–TiO_2	5.5-7.6	2.1	-42.4	[41]	2023	Composites & nanocomposites	4
3	NSC/Co/Cu	13.6-17.4	3.8	-51.6	[42]	2022	Carbon	4.5
4	MOF-based Ni/C	9.1-14.7	5.6	-42.8	[43]	2023	Synthetic Metals	4
5	Fe_3O_4@SiO_2	12.8-17.8	5.0	-50.2	[44]	2023	Journal of Materials Science	2.4
6	$NiFe_2O_4$/C	12.8-18.0	5.2	-57	[45]	2025	Journal of Alloys and Compounds	2.2
7	CoFe/C	4.4-10.6	6.2	-61.6	[46]	2024	Nanoscale	2.1
8	CoFe@C	12.2-18.0	5.8	-54	[47]	2024	Ceramics International	2.8
9	Cu_9S_5/RGO	5.7-9.7	4.0	-51.9	[48]	2021	Carbon	1.9
10	PMMA@$Ti_3C_2T_x$@SiO_2	13.3-16.2	2.9	-58.08	[49]	2023	Nano research	2.3
11	Ni/C@Co/NC	12.0-17.8	5.8	-66.03	[50]	2025	Ceramics International	5
12	ZnO/TiO_2/$Ti_3C_2T_x$	13.9-16.1	2.2	-31.96	[51]	2024	Ceramics International	3
13	Fe_2O_3/polypyrole	6.6-9.8	3.2	-56.4	[52]	2025	Applied Nano Materials	2.5

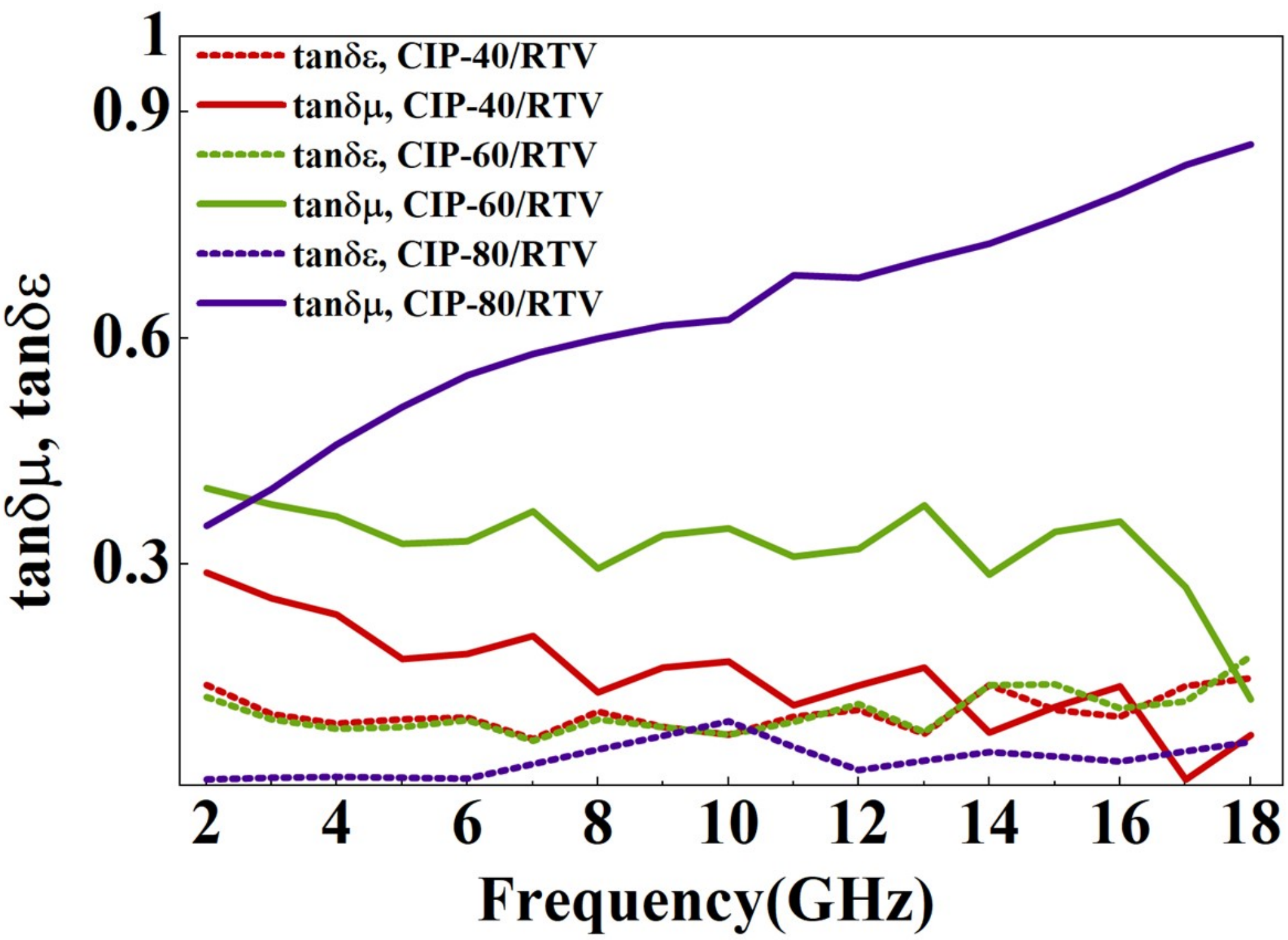

Fig. S7. Dielectric loss tangent (tan δε, dashed lines) and magnetic loss tangent (tan δμ, solid lines) of CIP-40/RTV, CIP-60/RTV, and CIP-80/RTV as a function of frequency over 2–18 GHz.
